# The Two PPX-GppA Homologues from *Mycobacterium tuberculosis* Have Distinct Biochemical Activities

**DOI:** 10.1371/journal.pone.0042561

**Published:** 2012-08-03

**Authors:** Mei Y. Choi, Ying Wang, Leo L. Y. Wong, Bing-tai Lu, Wen-yang Chen, Jian-Dong Huang, Julian A. Tanner, Rory M. Watt

**Affiliations:** 1 Oral Biosciences, Faculty of Dentistry, The University of Hong Kong, Prince Philip Dental Hospital, Sai Ying Pun, Hong Kong SAR, China; 2 Wuhan Centers for Disease Prevention and Control, Wuhan, Hubei Province, China; 3 Department of Anatomy, Li Ka Shing Faculty of Medicine, The University of Hong Kong, Pokfulam, Hong Kong SAR, China; 4 Department of Biochemistry, Li Ka Shing Faculty of Medicine, The University of Hong Kong, Pokfulam, Hong Kong SAR, China; University of Padova, Italy

## Abstract

Inorganic polyphosphate (poly-P), guanosine pentaphosphate (pppGpp) and guanosine tetraphosphate (ppGpp) are ubiquitous in bacteria. These molecules play a variety of important physiological roles associated with stress resistance, persistence, and virulence. In the bacterial pathogen *Mycobacterium tuberculosis*, the identities of the proteins responsible for the metabolism of polyphosphate and (p)ppGpp remain to be fully established. *M. tuberculosis* encodes two PPX-GppA homologues, Rv0496 (MTB-PPX1) and Rv1026, which share significant sequence similarity with bacterial exopolyphosphatase (PPX) and guanosine pentaphosphate 5′-phosphohydrolase (GPP) proteins. Here we delineate the respective biochemical activities of the Rv0496 and Rv1026 proteins and benchmark these against the activities of the PPX and GPP proteins from *Escherichia coli*. We demonstrate that Rv0496 functions as an exopolyphosphatase, showing a distinct preference for relatively short-chain poly-P substrates. In contrast, Rv1026 has no detectable exopolyphosphatase activities. Analogous to the *E. coli* PPX and GPP enzymes, the exopolyphosphatase activities of Rv0496 are inhibited by pppGpp and, to a lesser extent, by ppGpp alarmones, which are produced during the bacterial stringent response. However, neither Rv0496 nor Rv1026 have the ability to hydrolyze pppGpp to ppGpp; a reaction catalyzed by *E. coli* PPX and GPP. Both the Rv0496 and Rv1026 proteins have modest ATPase and to a lesser extent ADPase activities. pppGpp alarmones inhibit the ATPase activities of Rv1026 and, to a lesser extent, the ATPase activities of Rv0496. We conclude that PPX-GppA family proteins may not possess all the catalytic activities implied by their name and may play distinct biochemical roles involved in polyphosphate and (p)ppGpp metabolic pathways.

## Introduction

All living cells appear to have the physiological ability to synthesize and degrade inorganic polyphosphate (poly-P) molecules. These linear biopolymers comprise chains of phosphate (Pi) residues linked via ‘high-energy’ phosphoanhydride bonds, and range from a few to several hundred phosphate residues in length. In bacterial systems, poly-P is involved in a diverse range of biochemical, physicochemical and biological processes; e.g. the modulation of membrane structure and permeability; cell morphogenesis; DNA replication; as well as RNA and protein degradation. Poly-P also acts as an intracellular phosphate store and a biochemical phosphorylation agent (reviewed in refs. [1]–[4]). Within pathogenic species of bacteria, polyphosphate has been associated with enhanced levels of virulence, motility, stationary phase survival, persistence, resistance to complement-mediated cell lysis and increased biofilm formation [1]–[10]. Bacterial polyphosphate metabolism is also of notable environmental importance, playing a key role in the biological removal of phosphate from wastewater [11]. Consequently, the modulation of intracellular poly-P concentrations is of pivotal importance to numerous physiological processes involved in bacterial growth, viability, adaptability and infection.

The metabolism of poly-P in bacteria is mediated by several highly-conserved protein families, including: polyphosphate kinase 1 (PPK1), the main poly-P synthesizing enzyme in most species; polyphosphate kinase 2 (PPK2); polyphosphate/ATP NAD kinase (PpnK/NADK); polyphosphate-AMP phosphotransferase (PAP); polyphosphate glucokinase (GK); and exopolyphosphatase (PPX), the main hydrolytic enzyme in most species [1]–[4]. PPX proteins processively cleave phosphate residues from the termini of the polyphosphate chains [12]–[15]. In *Escherichia coli*, the guanosine pentaphosphate 5′-phosphohydrolase (GPP, GppA) enzyme also has strong exopolyphosphatase activities [16]. The primary function of this enzyme is to remove the terminal 5′-phosphate from guanosine 5′-triphosphate, 3′-diphosphate (guanosine pentaphosphate, pppGpp), to form guanosine 3′,5′-bisdiphosphate (guanosine tetraphosphate, ppGpp) [17]. Collectively referred to as (p)ppGpp, these two small molecule ‘alarmones’ are key players in the bacterial stringent response, a coordinated physiological process that enables bacteria to conserve and recycle resources during periods of environmental stress or nutritional deficiency [18]–[21]. In *E. coli*, it has been demonstrated that pppGpp, and to a lesser extent ppGpp, inhibit the exopolyphosphatase activities of the PPX enzyme, thereby promoting the intracellular accumulation of poly-P [22]. This single finding has led to the general paradigm that (p)ppGpp molecules inhibit the activities of PPX enzymes throughout bacteria [1], [2], [23].

Members of the Actinobacteria, (e.g. mycobacteria, corynebacteria, actinomycetes) lack identifiable PPX or GPP proteins. Instead, they generally encode two ‘PPX-GppA’ family proteins (pfam02541) ca. 300–350 aa in length, which share 20–35% aa identity with each other, and share homology with both PPX and GPP proteins [10], [24]–[28] (see Figure S1). There is a dearth of biochemical data for ‘PPX-GppA’ enzymes, which are generally assumed to have the ability to hydrolyze both polyphosphate and pppGpp substrates. This prompted us to comprehensively investigate the activities of the two PPX-GppA homologues encoded by the bacterial pathogen *Mycobacterium tuberculosis*: Rv0496 and Rv1026, which share ca. 27% amino acid identity. Polyphosphate and (p)ppGpp metabolism have been relatively well-studied within this organism at both the biochemical and biological levels [8]–[10], [21], [29]–[39]. Evidence indicates that polyphosphate and the stringent response both appear to play pivotal roles in the ability of this organism to form virulent or persistent infections. However, many fundamental questions relating to the putative interplay between these two processes remain to be established.

Here we show that the Rv0496 (MTB-PPX1) protein functions as a short-chain exopolyphosphatase, whose activities are inhibited by (p)ppGpp alarmones. Most notably, neither MTB-PPX1 nor Rv1026 have the ability to hydrolyse pppGpp to ppGpp. Our results indicate that these two PPX-GppA protein homologues possess notably different biochemical activities.

## Results

### Rv0496 (MTB-PPX1) has exopolyphosphatase activities

**Figure 1:**
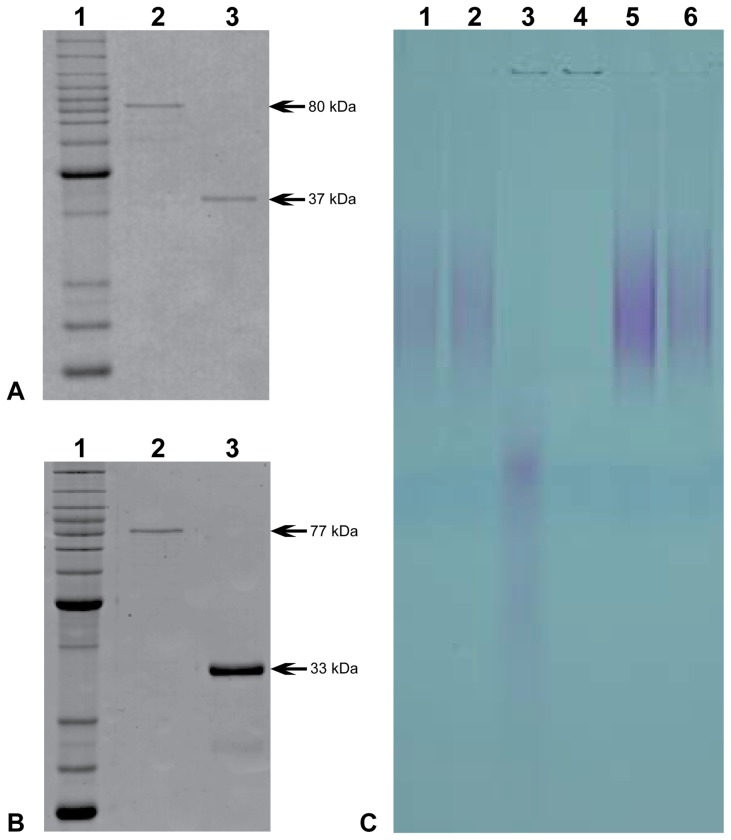
Purification and exopolyphosphatase activities of MTB-PPX1 and Rv1026. ***Panel A***: SDS-polyacrylamide gel of purified recombinant MTB-PPX1 (Rv0496) protein. *Lane 1*: protein ladder (BenchMark Protein from Invitrogen); *lane 2*, N-terminal Maltose Binding Protein (MBP)-MTB-PPX1 fusion protein (predicted molecular weight of 80 kDa); *lane 3*, untagged MTB-PPX1 (37 kDa; MBP-fusion removed using Factor Xa protease). ***Panel B***: SDS-polyacrylamide gel of purified recombinant Rv1026 protein. *Lane 1*: protein ladder; *lane 2*: MBP-Rv1026 (77 kDa); *lane 3*: untagged Rv1026 (33 kDa). ***Panel C***. Poly-P polyacrylamide gel showing exopolyphosphatase activities of MBP, MTB-PPX1, Rv1026 and *E. coli* GPP proteins. Reaction mixtures (100 *µ*l) containing protein (5 *µ*g), poly-P_130_ (0.1 mM), KCl (25 mM), with/without MnCl_2_ (1 mM) in HEPES buffer (50 mM, pH 6.8), were incubated for 1 hour at 37°C and analyzed on TBE 12% polyacrylamide gels. *Lane 1*: maltose binding protein (MBP; negative control); *lane 2*: MTB-PPX1, reaction without MnCl_2_; *lane 3*: MTB-PPX1; *lane 4*: *E. coli* GPP (EC-GPP); *lane 5*: Rv1026; *lane 6*: no protein added.

The *rv0496* and *rv1026* genes encoded by the H37Rv strain of *M. tuberculosis* were cloned into pMAL-c2 expression vectors, and over-expressed in *E. coli*. Milligram quantities of the respective N-terminal Maltose Binding Protein (MBP) fusion proteins were subsequently isolated in ca. 90–95% purity after one-step affinity-purification on amylose resin. The MBP-fusions (ca. 42 kDa) were cleaved using the Factor Xa protease, and the untagged recombinant Rv0496 and Rv1026 proteins were purified to >95% homogeneity using gel-filtration chromatography (Figure 1, Panels A and B). As the catalytic activities of the MBP-tagged and untagged forms of Rv0496 and Rv0126 were indistinguishable from one another; we used the tagged forms for the determination of their enzymatic kinetic parameters (see below), as they had slightly better *in vitro* stabilities (data not shown). We also cloned, expressed and purified the previously characterized *E. coli* PPX (EC-PPX) [12] and GPP (EC-GPP, GppA) [16] proteins, so that they could be included as side-by-side controls in the relevant assays (Supplementary Figure 2, Panels A and B). The multimeric states of the four recombinant proteins were determined by two independent approaches: light scattering and gel-filtration chromatography. As may be seen in Supplementary Figure 2D, consistent results were obtained for all four proteins using both approaches; namely they all adopted stable dimeric arrangements in solution. The dimeric arrangements of the *E. coli* PPX and GPP proteins established here is in agreement with previous reports [12], [16], as well as the X-ray crystal structures previously obtained for the *E. coli* PPX protein [14], [15].

A spectrophotometric phosphate release assay was used to determine the exopolyphosphatase activities of the Rv0496 and Rv1026 proteins, using EC-PPX and EC-GPP as positive controls. This assay was analogously used to determine the activities of the Cg0488 (CG-PPX1) and Cg1115 (CG-PPX2) proteins from *Corynebacterium glutamicum* [24]. A range of different buffers, salt concentrations and divalent metal ions were surveyed for their ability to promote the catalytic activities of the Rv0496 and Rv1026. It was subsequently found that the Rv0496 protein catalyzed the hydrolysis of polyphosphate in the presence of Mn^2+^ ions; and to a lesser extent, Mg^2+^ and Zn^2+^ ions; but no activity was detected in the presence of Ca^2+^, Co^2+^, Cu^2+^ or Fe^2+^ ions. Exopolyphosphatase activities were highest in HEPES buffer (50 mM, pH 6.8) containing 1 mM MnCl_2_ and 25 mM KCl (data not shown). No significant exopolyphosphatase activities could be detected for the recombinant Rv1026 protein under any condition tested. The respective abilities of the Rv1026 and Rv0496 proteins to catalyze the hydrolysis of a representative polyphosphate substrate (poly-P_130_) in the presence and absence of 1 mM MnCl_2_, may be seen in the poly-P PAGE gel shown in Figure 1C. It may be seen that he Rv0496 protein hydrolyzed the poly-P_130_ to shorter chain length polyphosphate (and phosphate) only in the presence of Mn^2+^ ions (compare lanes 2 and 3). We confirmed that there was no detectable polyphosphate hydrolysis activities in the absence of protein (lane 6), or in the presence of the purified maltose binding protein (lane 1). Under the same conditions, the *E. coli* GPP (EC-GPP) protein hydrolyzed the poly-P_130_ to undetectable levels. Consistent with the results from the spectrophotometric assays, the Rv1026 protein had no detectable activities (lane 5). We therefore renamed the Rv0496 protein ‘MTB-PPX1’, analogous to the *C. glutamicum* nomenclature [24]. However, we thought it inappropriate to refer to Rv1026 as ‘MTB-PPX2’, as this activity was not demonstrated.

**Figure 2:**
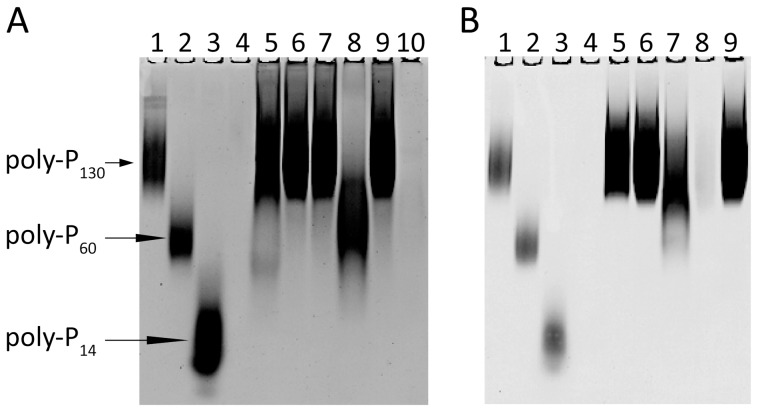
Hydrolysis of polyphosphate (poly-P_130_) mediated by cell free extracts of *Escherichia coli* CF6032 (*ΔgppA Δppkx*) and *Mycobacterium smegmatis* mc^2^155, supplemented by MTB-PPX1, Rv1026 or *E. coli* PPX proteins. Crude cell lysates were prepared from of cultures of *E. coli* MG1655 (wild type), *E. coli* CF6032 (triple mutant defective for GPP, PPX and PPK expression) and *M. smegmatis* mc^2^155 (see materials and methods). Cell lysate (2 *µ*g total protein), polyphosphate substrate (poly-P_130_, 0.1 mM) and the MTB-PPX1, Rv1026, *E. coli* PPX (EC-PPX), or maltose binding protein (MBP, negative control) proteins (2 *µ*g) were incubated at 37°C for 2 hours in 50 mM HEPES pH 6.8, 25 mM KCl, 1 mM MnCl_2_ (100 *µ*l). Reaction mixtures were resolved on TBE 12% polyacrylamide gels, and stained with toluidine blue, to analyze the polyphosphate products formed. Poly-P_60_ and poly-P_14_ (RegeneTiss) were included as polyphosphate chain length standards. **Panel A** *Lane 1*: poly-P_130_; *lane 2*: poly-P_60_; *lane 3*: poly-P_14_; *lane 4*: empty; *lane 5*: *E. coli* MG1655 lysate + poly-P_130_; *lane 6*: *E. coli* CF6032 lysate + poly-P_130_; *lane 7*: CF6032 + poly-P_130_ + MBP; *lane 8*: CF6032 + poly-P_130_ + MTB-PPX1, *lane 9*: CF6032 + poly-P_130_ + Rv1026; *lane 10*: CF6032+ poly-P_130_ + *E. coli* PPX (EC-PPX). **Panel B**. *Lane 1*: poly-P_130_ ; *lane 2*: poly-P_60_; *lane 3*: poly-P_14_; *lane 4*: empty; *lane 5*: *M. smegamtis* mc^2^155 lysate + poly-P_130_; *lane 6*: mc^2^155 + poly-P_130_ + MBP; *lane 7*: mc^2^155 + poly-P_130_ + MTB-PPX1, *lane 8*: mc^2^155 + poly-P_130_ + EC-PPX; *lane 9*: mc^2^155 + poly-P_130_ + Rv1026.

To confirm the results from these assays within a more biologically-relevant environment, we performed a set of complementation experiments using a crude cell lysate prepared from the CF6032 (*ΔgppA Δppkx*) mutant strain of *E. coli*, analogous to those previously performed by Kuroda *et al.* [22]. This triple mutant strain is defective for GPP, PPK and PPX protein expression, and hence lacks the ability to hydrolyze polyphosphate or pppGpp molecules to any significant extent. Recombinant MTB-PPX1 (Rv0496), Rv1026, *E. coli* PPX (EC-PPX) or MBP (negative control) proteins were added to a buffered reaction mixture containing cell lysate and poly-P_130_. After incubation at 37°C for 2 hours, polyphosphate content was analyzed on polacrylamide gels (see Figure 2, Panel A). It may be seen that the CF6032 lysate supplemented with MTB-PPX1 protein (lane 8) effectively mediated the hydrolysis of poly-P_130_ to shorter chain products (and phosphate); analogous to the results previously obtained using the fully-defined *in vitro* conditions (Figure 1 Panel C, lane 3). Under similar conditions, the EC-PPX protein (lane 10) hydrolyzed poly-P_130_ to undetectable levels, with no apparent formation of intermediate chain length products. In contrast, neither the MBP (lane 7) nor Rv1026 (lane 9) proteins were able to complement the CF6032 lysate for polyphosphate hydrolysis activity. A lysate that was analogously prepared from the wild type MG1655 strain of *E. coli* digested a significant proportion of the poly-P_130_ under equivalent conditions (lane 5) in the absence of added GPP or PPX protein, unlike the mutant strain (lane 6).

These assays were repeated using a crude cell lysate analogously prepared from cultured *Mycobacterium smegmatis* mc^2^155 cells (Figure 2, Panel B). As may be seen in lane 5, the *M. smegmatis* lysate did not possess detectable polyphosphate hydrolase activities. Similar to what was observed for the assays containing *E. coli ΔgppA Δppkx* lysate, the addition of the MTB-PPX1 protein resulted in a significant proportion of the poly-P_130_ being hydrolyzed to shorter chain length polyphosphate (lane 7). The consistent observation of significant amounts of (partially-digested) polyphosphate molecules of intermediate chain length (ca. 60–100 phosphate residues) in these assays indicates that Rv0496 does not function as a highly processive exopolyphosphatase, unlike EC-PPX and EC-GPP [12], [16]. Consistent with previous results, the addition of Rv1026 to the *M. smegmatis* lysate led to no detectable alterations in polyphosphate hydrolysis levels (lane 9). This further indicated that there were no components present in the *M. smegmatis* lysate that were able to stimulate or otherwise interact with the Rv1026 protein, to promote the hydrolysis of polyphosphate molecules.

### Rv0496 (MTB-PPX1) prefers short-chain length polyphosphates as substrates

The substrate specificities and kinetic parameters of the MTB-PPX1 (Rv0496) protein were probed in detail, using synthetic polyphosphate molecules of various chain lengths (poly-P_14_, poly-P_60_, poly-P_130_). The *K_m_* and *k_cat_* values determined for each of the three poly-P chain lengths are shown in Table 1. The *K_m_* (poly-P_14_) for MTB-PPX1 was 5.9±0.3 *µ*M, which was ca. two-fold lower than the *K_m_* for poly-P_60_, and ca. 3 times lower than the *K_m_* for poly-P_130_. The rate of hydrolysis for the three polyphosphate chain lengths followed a similar trend, with poly-P_14_ being hydrolyzed most rapidly (*k_cat_* = 10.2±0.1 s^−1^). This clearly indicated that MTB-PPX1 preferentially hydrolyzed short-chain poly-P molecules. Contrastingly, results from analogous experiments revealed that both the EC-GPP and EC-PPX proteins bound long-chain polyphosphate substrates most effectively. The *K_m_* (poly-P_130_) values for the EC-GPP and EC-PPX proteins were 3.2±1.1 *µ*M and 1.1±0.3 *µ*M, respectively, which is reasonably consistent with previous findings [12], [16]. However, we found that the *E. coli* PPX and GPP proteins hydrolyzed the shorter-chain poly-P_14_ and poly-P_60_ substrates significantly more rapidly than poly-P_130_, even though these substrates had higher *K_m_* values. The turnover numbers and catalytic efficiencies of EC-GPP and EC-PPX were notably higher than those of MTB-PPX1 (see Table 1), clearly indicating that the two *E. coli* enzymes had substantially higher exopolyphosphatase activities under the conditions tested.

**Table 1:** Kinetic parameters for the exopolyphosphatase activities of MTB-PPX1, EC-GPP and EC-PPX.

Protein		Poly-P_14_	Poly-P_60_	Poly-P_130_
**MTB-PPX 1**	***K_m_*** ** (μM)**	5.9±0.3	12.1±1.6	17.3±1.6
	***V_max_*** ** (μmol min^−1^ mg^−1^ of protein)**	11.0±0.1	7.9±0.2	6.6±0.2
	***k_cat_*** ** (s^−1^)**	10.2±0.1	7.3±0.2	6.7±0.2
	***k_cat_/K_m_*** ** (mM^−1^ s^−1^)**	1729±103	603±94	387±46
**EC-GPP**	***K_m_*** ** (μM)**	9.7±1.9	10.0±1.2	3.2±1.1
	***V_max_*** ** (μmol min^−1^ mg^−1^ of protein)**	22.9±1.5	17.6±0.6	9.2±0.5
	***k_cat_*** ** (s^−1^)**	18.3±1.2	14.1±0.5	7.4±0.4
	***k_cat_/K_m_*** ** (mM^−1^ s^−1^)**	1886±492	1410±219	2313±918
**EC-PPX**	***K_m_*** ** (μM)**	8.2±2.3	2.0±0.1	1.1±0.3
	***V_max_*** ** (μmol min^−1^ mg^−1^ of protein)**	19.3±1.8	14.1±1.2	9.3±0.6
	***k_cat_*** ** (s^−1^)**	16.7±1.6	12.2±1.0	8.1±0.5
	***k_cat_/K_m_*** ** (mM^−1^ s^−1^)**	2037±765	6100±793	7364±2452

Individual kinetic parameters (mean **v**alues ± S.D.) for the hydrolysis of three polyphosphate substrates with differing chain lengths (poly-P_14_, poly-P_60_, poly-P_130_) are shown for each enzyme, incorporating results from sets of assays performed in quadruplicate.

### Rv0496 and Rv1026 do not hydrolyze guanosine pentaphosphate (pppGpp)

To the best of our knowledge, there is no commercial supplier of pppGpp. Previous investigations have used a promiscuous purine nucleotide pyrophosphotransferase from *Streptomyces morookaensis* [40] or a purified *E. coli* ribosomal fraction that contains the RelA protein [40] to convert ATP + GTP into pppGpp. We have found that the RelQ protein homologue from *Enterococcus faecalis* (EF-RelQ, EFFG_01794) has excellent (p)ppGpp synthesis activities, with negligible (p)ppGpp hydrolysis activities (Choi *et al*., manuscript in preparation). Consequently, purified recombinant EF-RelQ protein (Figure S2 Panel C) [41], was used to enzymatically synthesize pppGpp and ppGpp from ATP + GTP, or from ATP + GDP, respectively. Anion exchange chromatography was used to separate the synthesized (p)ppGpp alarmones from the starting materials and AMP biproduct (see Figure S3) . The purified pppGpp and ppGpp nucleotides were then desalted on Sephadex G10 resin [42] and characterized as described by Hardiman *et al.* [43] to confirm their identity and purify.

**Figure 3:**
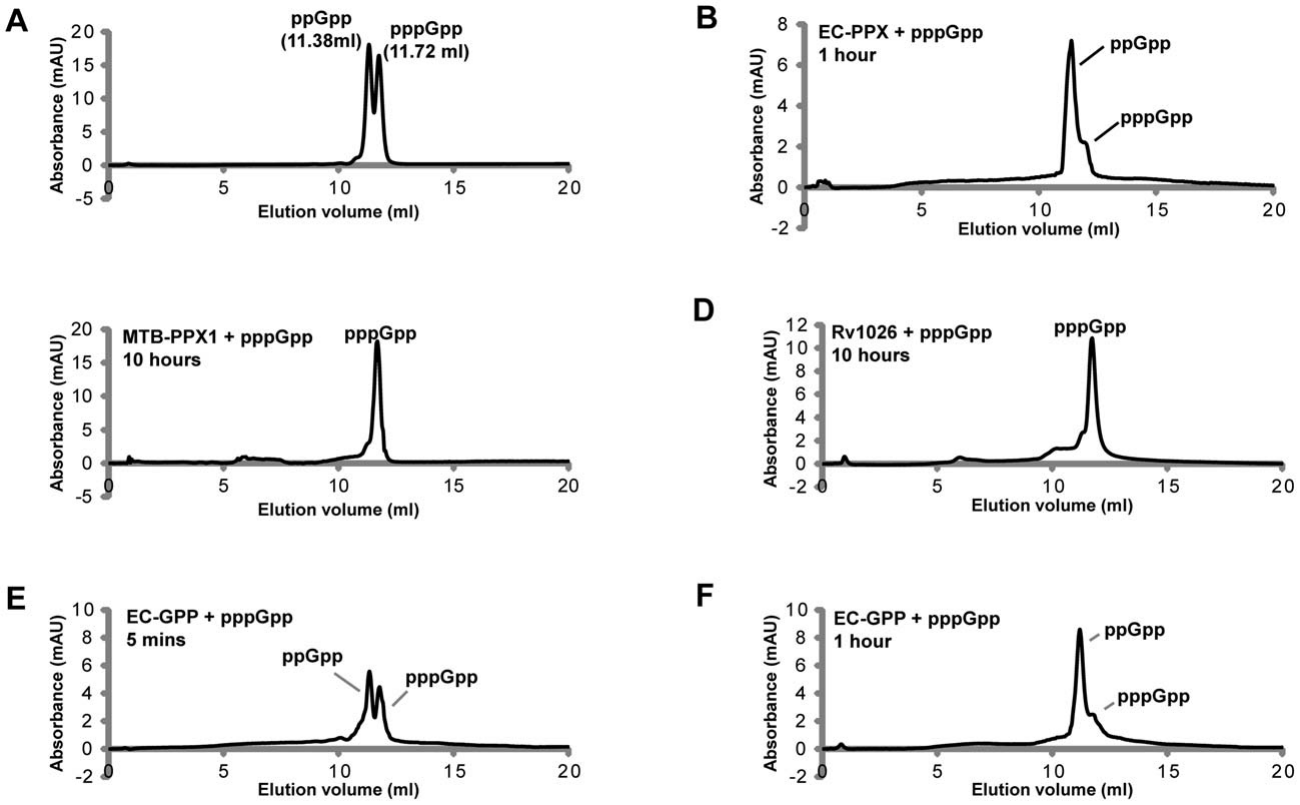
pppGpp hydrolysis activities of MTB-PPX1, Rv1026, *E. coli* GPP and *E. coli* PPX. Reaction mixtures (100 μl) containing 2 μg protein (MTB-PPX1, Rv1026, *E. coli* GPP or *E. coli* PPX), 0.1 mM pppGpp, 1 mM MnCl_2_ and 0.5 mM DTT in Tris-HCl buffer (25 mM, pH 7.4) were incubated at 30°C for the time indicated. Product mixtures were directly analyzed by anion exchange chromatography. Chromatograms obtained are shown in Panels A–F (y-axis: UV absorption, 254 nm; x-axis: elution volume). Under these conditions, pppGpp elutes at 11.72 ml and ppGpp elutes at 11.38 ml. ***Panel A***: mixture of pppGpp and ppGpp standards (0.1 mM of each); ***Panel B***: *E. coli* PPX (EC-PPX) + pppGpp, 1 hour incubation; ***Panel C***: MTB-PPX1 + pppGpp, 10 hours incubation; ***Panel D***: Rv1026 + pppGpp, 10 hours incubation; **Panel E**: *E. coli* GPP (EC-GPP) + pppGpp, 5 minutes incubation; ***Panel F***: EC-GPP + pppGpp, 1 hour incubation.

As previous reports have failed to investigate the putative guanosine pentaphosphate 5′-phosphohydrolase activities of the PPX-Gppa homologues from *A. aeolicus* and *C. glutamicum* [24], [26], [27], we felt it was highly-important to determine whether the MTB-PPX1 (Rv0496) or Rv1026 proteins had this catalytic ability. As the *E. coli* GPP and PPX appear to be the only proteins clearly demonstrated to have this activity, they were both included as positive controls [16], [22]. Levels of pppGpp and ppGpp present after prolonged enzymatic incubation with MTB-PPX1 or Rv1026 were analyzed using anion exchange chromatography (see Figure 3). Under the conditions used, ppGpp and pppGpp had distinct retention times (eluting at 11.38 ml and 11.72 ml, respectively). Whilst both the EC-GPP and EC-PPX proteins effectively cleaved the 5′-terminal phosphate group, the MTB-PPX1 and Rv1026 proteins completely lacked this activity under all conditions employed (see Figure 3).

To further confirm the pppGpp hydrolase abilities of the MTB-PPX1 (Rv0496) and Rv1026 proteins, we performed sets of complementation assays using crude cell lysates prepared from *E. coli* MG1655 (wild type), CF6032 (*ΔgppA Δppkx*) and *M. smegmatis* mc^2^155, analogous to those used to analyze their exopolyphosphatase activities. In these assays, 2 μg of purified protein and cell lysate (2 μg total protein) were incubated with pppGpp (0.1 mM) in buffered reaction mixtures. The pppGpp and ppGpp levels were then quantified using anion exchange chromatography. The chromatograms obtained are shown in Figures S4, S5 and S6, respectively. As may be seen in the chromatogram shown in Figure S4 Panel A, more than 80% of the pppGpp was hydrolyzed to ppGpp by the wild type *E. coli* MG1655 lysate. In contrast, there was no significant hydrolysis of pppGpp mediated by the *E. coli* CF6032 or *M. smegmatis* lysates under the conditions used (Panels A; in Figures S5 and S6). When either the EC-PPX or EC-GPP proteins were added, there was near-complete conversion of pppGpp to ppGpp in all three cell lysate mixtures (Panels C; Figures S4, S5, S6). In contrast, when the MTB-PPX1, Rv1026 or MBP proteins were added to the lysates under identical conditions, there was no detectable change in pppGpp or ppGpp levels, indicative of the complete absence of pppGpp hydrolase activity.

### MTB-PPX1 and Rv1026 hydrolyze ATP and ADP substrates, but lack GTPase activities

**Figure 4:**
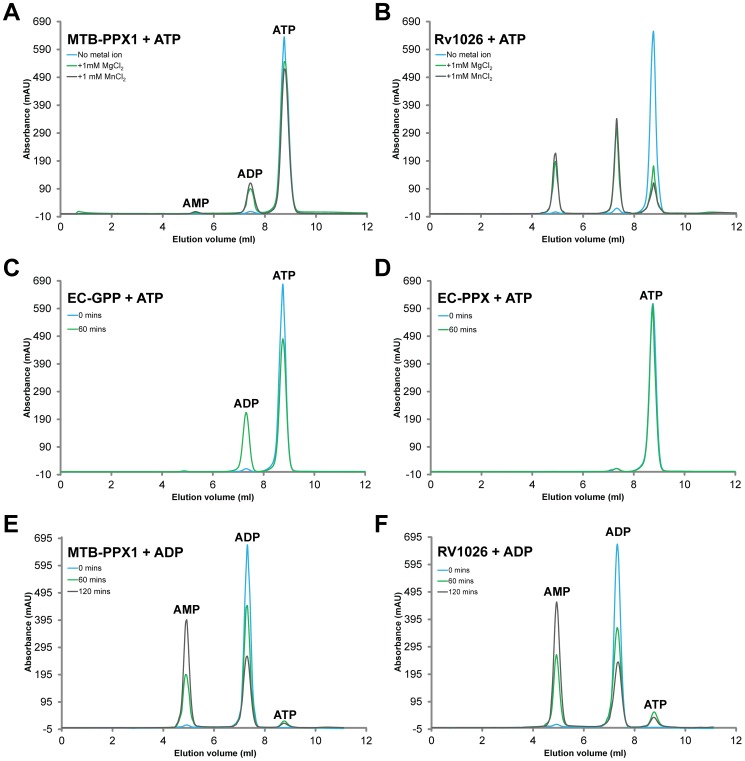
ATPase and ADPase activities of MTB-PPX1 and Rv1026. Reaction mixtures (100 μl) containing 1 μg protein (MTB-PPX1, Rv1026, *E. coli* GPP or *E. coli* PPX), 1 mM ATP or ADP, 4 mM (NH_4_)_2_SO_4_, in Tris-HCl buffer (50 mM, pH 7.4), with or without 1 mM MnCl_2_ or MgCl_2_; were incubated at 30°C for 0, 60 or 120 minutes (as indicated). Product mixtures were analyzed using anion exchange chromatography, with chromatograms shown in Panels A–F. ***Panel A*** *:* MTB-PPX1 + ATP; no added metal ion (blue line), with 1 mM MgCl_2_ (green line), with 1 mM MnCl_2_ (black line); incubated for 60 minutes. ***Panel B***: Rv1026 + ATP (as for Panel A). ***Panel C***: *E. coli* GPP (EC-GPP) + ATP + MnCl_2_: 0 minutes incubation (blue line), 60 minutes incubation (green line). ***Panel D***: *E. coli* PPX (EC-PPX) + ATP + MnCl_2_ (as for Panel C). ***Panel E***: MTB-PPX1 + ADP + MnCl_2_; 0 minutes (blue line), 60 minutes (green line), 120 minutes (black line). ***Panel F***: Rv1026 + ADP + MnCl_2_ (as for Panel E).

The NTP and NDP hydrolytic activities of MTB-PPX1 and Rv1026 were surveyed, to investigate their substrate specificities. The Rv1026 and MTB-PPX1 proteins were both found to possess modest ATPase activities (Figure 4, Panels A and B), which were optimal at ca. pH 7.4 and were dependent on Mg^2+^ or Mn^2+^ ions (above ca. 0.5–1 mM). The kinetic parameters established for MTB-PPX1 were: *K_m (ATP)_ = *6.1±0.8 mM; *V_max_* = 0.7±0.1 μmol min^−1^ mg^−1^ protein; *k_cat_* = 1.9±0.3 s^−1^; *K_m (ATP)_*/*k_cat_* = 0.31±0.09 mM^−1^s^−1^. For the Rv1026 protein: *K_m (ATP)_ = *2.6±1.0 mM; *V_max_* = 0.7±0.1 μmol min^−1^ mg^−1^ protein; *k_cat_* = 4.4±0.6 s^−1^; *K_m (ATP)_*/*k_cat_* = 1.69±0.87 mM^−1^s^−1^. These data clearly indicated that the Rv1026 was a more active and efficient ATPase than MTB-PPX1. Both proteins could also catalyze the hydrolysis of ADP to AMP (Figure 4, Panels E and F), with Rv1026 being slightly more active than MTB-PPX1 in this regard. As may be seen in Panels A and B, the Rv1026 protein mediated the sequential hydrolysis of ATP to ADP to AMP under the conditions employed; clearly indicating it was more efficient ATPase and ADPase than MTB-PPX1. The MTB-PPX1 and Rv1026 enzymes also had the ability to synthesize small amounts of ATP from ADP (see Figure 4, Panels E and F). From this observation, we speculate that there may a phosphory transfer to an active site residue. If this phosphorylated enzyme intermediate is sufficiently long-lived; after the release of the initial AMP product, there could be subsequent binding and phosphorylation of a second ADP molecule, resulting in the synthesis of ATP.

**Figure 5:**
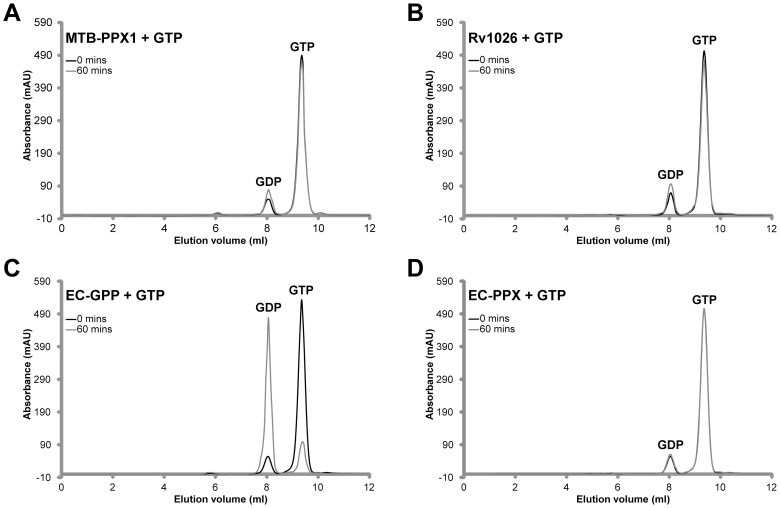
GTPase activities of MTB-PPPX1, Rv1026, *E. coli* GPP and *E. coli* PPX. Reaction mixtures (100 μl) containing 1 μg of protein (MTB-PPX1, Rv1026, *E. coli* GPP (EC-GPP) or *E. coli* PPX (EC-PPX)), 1 mM GTP, 1 mM MnCl_2_, 4 mM (NH_4_)_2_SO_4_, in 50 mM Tris-HCl buffer (50 mM, pH 7.4), were incubated at 37°C for 0 or 60 minutes. Product mixtures were directly analyzed using anion exchange chromatography. Chromatograms obtained are shown in Panels A–D. ***Panel A***: MTB-PPX and GTP. ***Panel B***: Rv1026 and GTP. ***Panel C***: EC-GPP and GTP. ***Panel D***: EC-PPX and GTP.

The MTB-PPX1 and Rv1026 proteins possessed extremely low GTPase activities, which were only marginally above background (non-enzymatic) levels under any of the conditions tested (Figure 5). Notably, the addition of polyphosphate or phosphate ions appeared to have no stimulatory or inhibitory effects on the ATPase, ADPase or GTPase activities of either enzyme; and neither poly-P nor phosphate acted as co-substrates (data not shown). Furthermore, whilst the *E. coli* GPP protein was a highly efficient GTPase and a modest ATPase, neither of these activities could be detected for the *E. coli* PPX protein (see Figures 4 and 5).

### (p)ppGpp alamones inhibit the exopolyphosphatase activities of MTB-PPX1

As the MTB-PPX1 and Rv1026 proteins lacked the ability to hydrolyze pppGpp to ppGpp, we next investigated whether their exopolyphosphatase were inhibited by either of these alarmone molecules. The *E. coli* PPX and GPP proteins were included as positive controls, as Kuroda *et al.* have previously shown that pppGpp, and to a lesser extent ppGpp, inhibit their poly-P hydrolysis activities [22]. The *K_i_* values reported for *E. coli* PPX were 10 μM for pppGpp, and 200 μM for ppGpp. We consequently used 1 mM concentrations of either pppGpp or ppGpp in our inhibition experiments in order to obtain definitive results. In comparative experiments, we quantified the maximal rate of poly-P_130_ hydrolysis (*V_max_*) by the EC-GPP, EC-PPX and MTB-PPX-1 enzymes in the presence of pppGpp or ppGpp, versus alarmone-free controls. Results are summarized in Table 2A. Consistent with the findings of Kuroda *et al.* [22], pppGpp inhibited the exopolyphosphatase activities of EC-PPX ca. 10-fold more effectively than ppGpp. Analogous results were obtained for the EC-GPP protein, with pppGpp inhibiting poly-P hydrolysis 5-fold more effectively than ppGpp. Similarly, we found that the maximal rate of poly-P_130_ hydrolysis by the MTB-PPX1 protein was reduced by ca. 38% in the presence of 1 mM pppGpp. Under the same conditions, 1 mM ppGpp lowered the rate of poly-P hydrolysis by only ca 6% (approximately 6-fold less effectively). In contrast, the exopolyphosphatase activities of MTB-PPX1 were not significantly affected by 5′-mono-, di- and triphosphates of adenosine or guanosine, even at 5 mM concentrations (data not shown). Taken together, these experiments revealed that the pppGpp and ppGpp alarmones had analogous inhibitory effects on the exopolyphosphatase activities of the *E. coli* GPP, PPX and MTB-PPX1 proteins; with pppGpp being a significantly more potent inhibitor.

**Table 2:** Inhibition of Exopolyphosphatase and ATPase activities by pppGpp and ppGpp.

A			
Protein	Exopolyphosphatase activities		
	*V_max_* (μM/min)		
	No (p)ppGpp	+1 mM pppGpp	+1 mM ppGpp
		(% decrease)	(% decrease)
**MTB-PPX1**	43.8±3.8	27.2±2.5	41.2±1.3
		(37.9±6.7%)	(5.9±0.7%)
**EC-GPP**	115.2±12.5	81.2±3.8	108.3±3.4
		(29.5±4.4%)	(6.0±0.5%)
**EC-PPX**	151.7±15.2	15.8±2.5	135.3±2.1
		(89.6±20.1%)	(10.8±1.2%)

B			
Protein	ATPase activities		
	*V_max_* (μM/min)		
	No (p)ppGpp	+1 mM pppGpp	+1 mM ppGpp
		(% decrease)	(% decrease)
**MTB-PPX1**	2.4±0.1	2.2±0.9	2.5±0.2
		(8.3±4.1%)	(−4.1±0.3%)
**Rv1026**	6.8±1.1	4.6±0.6	6.9±0.9
		(32.3±9.4%)	(−1.5±2.0%)

The maximum enzymatic reaction rates (*V_max_* values) calculated for assays performed in the presence of 1 mM pppGpp or ppGpp, were compared with the *V_max_* values obtained from analogous assays performed in the absence of (p)ppGpp. The percentage decrease in *V_max_* values are reported in parentheses. *V_max_* values are reported as the mean value (μM/min) ± S.D.

### (p)ppGpp alamones inhibit the ATPase activities of Rv1026 and MTB-PPX1

We also investigated whether the pppGpp or ppGpp alarmones could inhibit the ATPase activities of the MTB-PPX1 or Rv1026 proteins. 1 mM pppGpp inhibited the ATPase activities of Rv1026 (*V_max_* reduced by ca. 32%), but ppGpp had no significant effects (see Table 2A). pppGpp molecules also inhibited the ATPase activities of the MTB-PPX1 protein under these conditions, but to a lesser extent (*V_max_* reduced by ca. 8%). ppGpp alarmones had no detectable inhibitory effects. Data obtained from further inhibition assays performed over a range of ATP concentrations, were consistent with pppGpp inhibiting the ATPase activities of Rv1026 in a competitive manner (see Table S2).

## Discussion

Here we show that the MTB-PPX1 (Rv0496) protein functions as an exopolyphosphatase, exhibiting a distinct preference for relatively short-chain polyphosphate substrates. Its activities appear to be similar to those of the CG-PPX2 (Cg1115) protein from *C. glutamicum* [24]. Both enzymes prefer short-chain polyphosphate substrates, require Mg^2+^ or Mn^2+^ ion cofactors, and have optimal activities at slightly acidic pH values (pH 6.8). The sequences of the MTB-PPX1, Rv1026 and CG-PPX2 proteins align with the N-terminal regions of the *E. coli* PPX and GPP proteins (Figure S1). The available structural and bioinformatic data indicates that polyphosphate and pppGpp substrates are hydrolyzed at a single active site located within this region [14], [15], [24], [26], [27]. Both the MTB-PPX1 and Rv1026 proteins contain conserved amino acid residues implicated in the catalytic mechanism. Asp135 and Glu142 (MTB-PPX1) and Asp146 and Glu153 (Rv1026) are predicted to be responsible for binding the essentially-required Mn^2+^ or Mg^2+^ ions. Arg84 and Glu112 (MTB-PPX1) and Arg90 and Glu123 (Rv1026) are implicated in the hydrolysis of the phosphoanhydride bond linking the terminal and penultimate phosphate units in the poly-P or ATP (and ADP) substrates. X-ray crystal structures of the *E. coli* PPX protein suggest that the polyphosphate chain is bound primarily by residues located within the C-terminal region [14], [15]. This is supported by biochemical and biophysical data obtained from truncated forms of the EC-PPX protein lacking the C-terminal domain [13]. As these ca. 150–200 C-terminal residues are absent in MTB-PPX1 (and CG-PPX2), it most likely adopts an alternative mode of polyphosphate binding. This may be responsible for the differences in polyphosphate chain length preferences observed for MTB-PPX1, compared to the *E. coli* PPX and GPP proteins; which have higher affinities for short and long-chain polyphosphate substrates, respectively (Table 1). Bolesch and Keasling previously defined the N-terminal ca. 300 residues of EC-PPX as functioning as a ‘quasi-processive’ exopolyphosphatase [13]. This is what the MTB-PPX1 protein appears to function as. The activities of additional diverse PPX-GppA homologues will need to be characterized to determine whether this putative structure-function relationship generally holds true.

Our finding that the *E. coli* GPP protein has notable GTPase activities is not consistent with the previous report by Hara and Sy [17]. We tentatively speculate that these authors may have inadvertently purified and characterized the *E. coli* PPX protein; as their report predates the identification and characterization of EC-PPX [12]; and the discovery that this protein also possesses guanosine pentaphosphate 5′-phosphohydrolase activities [22]. Future investigations may substantiate or repudiate this speculation.

Transposon mutagenesis has previously shown that the *rv0496* gene was non-essential in the H37Rv strain, whilst *rv1026* was required for optimal growth [44]. Rv0496 (MTB-PPX1) was found to be secreted from *M. tuberculosis* cells, and was identified as a T-cell antigen with potential for vaccine development [45], [46]. Thayil *et al.* [10] have recently reported that the 344 aa MT0516 protein from *M. tuberculosis* CDC1551, which has an identical sequence to Rv0496 (MTB-PPX1) from the H37Rv strain, could hydrolyze a 65-mer of polyphosphate; noting that its polyphosphate hydrolase activities were not inhibited in the presence of 1 μM of ppGpp. The authors also reported that the intracellular poly-P levels were elevated in a MT0516-deficient strain of *M. tuberculosis* during the mid-logarithmic and late stationary growth phases. Using a guinea pig lung model, they further demonstrated that the activities of MT0516 played a key role in enabling *M. tuberculosis* cells to grow and persist within necrotic granulomas. The authors did not report the activities of the 319aa MT1054 protein, which has an identical amino acid sequence to the Rv1026 protein characterized here. Our results are broadly consistent with the preliminary biochemical data reported by Thayil *et al.*, and greatly extend upon it.

The PPK1 protein appears to be the major source of polyphosphate in *M. tuberculosis*, synthesizing a relatively broad spectrum of poly-P chains lengths, ranging from ca. 200 to 800-mers [3], [8], [30]. It should be noted that MTB-PPX1 is one of a number of proteins that possess the catalytic ability to catabolize polyphosphate molecules via hydrolysis or other biochemical processes. The PPK2 (Rv3232c) protein functions primarily as a polyphosphate-dependent ATP (and possibly GTP) regenerating enzyme, rather than a source of poly-P [4], [8], [30], [38], [39], [47]. Other net-consumers of poly-P are polyphosphate glucokinase (Rv2702) [34] and PpnK/NADK (Rv1695) [35]. The physiological significance of the MTB-PPX1 protein's preference for short-chain poly-P remains obscure, as the distributions of polyphosphate chain lengths in *M. tuberculosis* cells are not accurately known.

The biological role of the Rv1026 protein has not yet been established. Its expression was previously shown to be up-regulated in cells treated with translation-inhibitors [48], and it was also induced during intracellular growth within macrophages [49]. Our results reveal that Rv1026 lacks the ability to hydrolyze poly-P or pppGpp substrates. It possibly plays a role in adenine nucleotide equilibration; promoting the formation of ADP and AMP, at the expense of ATP (Figure 3). However, the physiological significance of these biochemical activities and the relevance of the pppGpp-mediated inhibition of its ATPase activities remain obscure. Shi *et al.* recently reported that the over-expression of Rv1026 in *M. smegmatis* led to a small, temporary decrease in the relative abundance of polyphosphate [25]. Our results suggest that the over-expression of Rv1026 may lower polyphosphate levels in *M. smegmatis* cells in an indirect manner. It would putatively reduce the polyphosphate-synthesizing activities of the *M. smegmatis* PPK1 protein, by lowering its substrate (ATP) levels and increasing it product (ADP)-mediated inhibition; amongst other possible pleiotropic effects [8], [30], [39], [50]. However, we cannot exclude the possibility that Rv1026 functions as an exopolyphosphatase when expressed within *M. tuberculosis* or other mycobacterial cells.

As mediators of the stringent response, pppGpp and ppGpp alarmones affect global changes in stable RNA levels, DNA synthesis, amino acid biosynthesis and protein degradation [18], [19], [20], [21]. The stringent response plays a pivotal role in the ability of *M. tuberculosis* cells to exist in a prolonged state of dormancy within macrophage phagosomes, and to form persistent and virulent infections [29], [31]. Our results clearly demonstrate that the both the MTB-PPX1 and Rv1026 proteins lack the ability to hydrolyze pppGpp to ppGpp (Figure 3). It remains to be seen whether *M. tuberculosis* encodes an alternative protein with GPP functionality, or does not require this alarmone-converting activity. The bifunctional Rel_MTB_ (Rv2583c) protein is the only source of pppGpp and ppGpp molecules in *M. tuberculosis* [32], [33]. Polyphosphate molecules modulate the transcription of *rel_MTB_* via a two-component MprAB/SigE pathway, thereby regulating (p)ppGpp production [8]. Via this mechanism, increased polyphosphate levels lead to increased (p)ppGpp levels. In *E. coli*, there is positive feedback via the (p)ppGpp-mediated inhibition of PPX activities [22]; thereby prolonging the intracellular lifetime of polyphosphate. As pppGpp, and to a lesser extent ppGpp, inhibit the exopolyphosphatase activities of MTB-PPX1 (Table 2A), our results suggest that this regulatory feedback is also present in *M. tuberculosis*.

To briefly conclude, our results demonstrate that the Rv0496 (MTB-PPX1) protein functions as a short-chain exopolyphosphatase, whose activities are inhibited by (p)ppGpp alarmones produced during the bacterial stringent response. Neither MTB-PPX1 nor Rv1026 have the ability to hydrolyze pppGpp, a property that makes them notably different to the GPP and PPX proteins from *E. coli*. The data presented here reveals that members of the PPX-GppA protein family possess notable differences in their catalytic activities, indicating that overall sequence homology may not be a reliable indicator of biochemical or biological functionality.

## Materials and Methods

### Gene cloning procedures

#### Rv0496 (MTB-PPX1) and Rv1026

The *rv0496* and *rv1026* genes were PCR amplified from *M. tuberculosis* H37Rv genomic DNA using the Rv0496for2 and Rv0496rev2, and Rv1026for and Rv1026rev primer pairs, respectively, with the use of LongAmp Taq DNA polymerase from New England Biolabs (NEB). After TOPO cloning (pCR2.1 TOPO-TA cloning kit from Invitrogen, Life Technologies), amplified genes were subcloned (XbaI/HindIII for *Rv0496*, BamHI/HindIII for *Rv1026*), into similarly digested pMAL-c2 expression vectors (NEB), to encode N-terminal maltose binding protein (MBP; MalE) fusions. The MBP protein was expressed from unmodified plasmid pMAL-c2, for use as a negative control. For a list of the primers used in this study, see Table S1.

#### EF-RelQ

The *EF-relQ* (*EFFG_01794*) gene was PCR amplified from *Enterococcus faecalis* ATCC 29212 genomic DNA using the EFRelQfor and EFRelQrev primers, TOPO cloned, then subcloned via BamHI/XhoI into pET28a(+) (Novagen, Merck4Biosciences).

#### EC-GPP and EC-PPX

The *gppa* (*b3779*) and *ppx (b2502)* genes were PCR amplified from *E. coli* BL21 (DE3) genomic DNA using the EcoliGppafor and EcoliGpparev, and EcoliPpxfor and EcoliPpxrev primers, respectively, TOPO cloned, then respectively subcloned into pET28a(+) via BamHI/XhoI.

### Protein expression and purification

*E. coli* BL21(DE3) cells (Invitrogen) transformed with the appropriate expression plasmid, were cultured at 37°C in Luria Bertani (LB) medium (USB Corp.) until the OD_600_ reached ca. 0.6. Protein expression was induced by adding (isopropyl β-D-1-thiogalactopyranoside, IPTG; GE Healthcare) to a final concentration of 0.3 mM, then cells were cultured at room temperature (ca. 25°C) for 4–6 hours. Cells pellet were collected and lysed by sonication (Vibra Cell, Sonics & Materials Inc.) in either ‘Ni-binding buffer’ (25 mM Tris-HCl pH 7.4, 500 mM NaCl, 20 mM imidazole) for pET28a(+) constructs; or ‘maltose-binding buffer’ (25 mM Tris-HCl pH 7.4, 200 mM NaCl, 10% glycerol) for pMAL-C2 constructs, containing protease inhibitors (Complete EDTA-free, Roche). Lysates were centrifuged (15,000×g, 60 min, 4°C), then supernatants were filtered (0.45 μm syringe filter, Iwaki) prior to purification onto a 5 ml HiTrap Chelating HP column (GE Healthcare; for pET28a(+) constructs) or 5 ml MBPTrap HP (GE Healthcare; for pMAL-C2 constructs). Recombinant *E. coli* GPP and PPX proteins were eluted with 25 mM Tris-HCl pH 7.4, 500 mM NaCl, 60 mM imidazole. EF-RelQ protein was eluted with 25 mM Tris-HCl pH 7.4, 500 mM NaCl, 100 mM imidazole. Rv0496 and Rv1026 proteins were eluted with maltose-binding buffer containing 10 mM maltose. The N-terminal maltose binding protein (MBP) fusion was cleaved using Factor Xa (Novagen) according to the manufacturer's protocol. Cleaved protein mixtures were dialyzed (4°C) against fresh maltose-binding buffer, then maltose affinity chromatography was used to remove the cleaved MBP tags. Protein concentrations were determined using the BioRad Protein assay (Bradford Reagent, BSA standard), and protein purity was determined by densitometry after 12% sodium dodecyl sulfate polyacrylamide gel electrophoresis (SDS–PAGE).

### Gel filtration chromatography

The molecular masses of the recombinant Rv0496 (MTB-PPX1), Rv1026, *E. coli* GPP, *E. coli* PPX and *E. faecalis* RelQ proteins were determined by size exclusion chromatography on a Superdex 200 gel filtration column (GE Healthcare) using an AKTA purifier system (GE Healthcare). Calibration curves were constructed using protein standards (HMW and LMW gel filtration calibration kits, GE Healthcare).

### Light scattering

Known dilutions of the purified protein samples (2 mg/ml, 1 mg/ml, 0.5 mg/ml; in 25 mM Tris-HCl pH 7.4, 25 mM NaCl) were pipetted onto 384-well Greiner Glass Bottom SensoPlates (GreinerBio-One, Inc.). Samples were irradiated using a semiconductor laser (830 nm), on a DynaPro Plate Reader Plus (Wyatt Technology Corp.). Collected data were analyzed using DynaPro dynamic light scattering instrument software (version 7.0.3.12; Wyatt Technology Corp.) to calculate the molecular mass.

### Exopolyphosphatase assays

The exopolyphosphatase activities of Rv0496 (MTB-PPX1), EC-PPX and EC-GPP were determined using continuous spectrophotometric assays, quantifying phosphate (Pi) release using the EnzChek phosphate assay kit (Invitrogen) according to the manufacturer's protocol; analogous to the method reported by Lindner *et al.* [24]. Unless otherwise stated, assays were performed in 96-well plates at 37°C, in 200 μl of 50 mM HEPES pH 6.8, 1 mM MnCl_2_, 25 mM KCl, containing EC-PPX, EC-PPX or Rv0496 (MTB-PPX1) protein (2 μg, 0.1–0.2 μM), polyphosphate (0–150 μM) of the stated chain length (poly-P_14_, poly-P_60_, poly-P_130_); quantifying changes in the absorbance at 360 nm using a Spectra Max 340 plate reader (Molecular devices). Inorganic polyphosphate samples with average chain lengths of P14, P60 and P130 were provided by Dr. T. Shiba (RegeneTiss Inc., Japan). *V_max_* and *K_m_* values were determined by fitting data to the Michaelis-Menten equation using Origin 6.0 (OriginLab). Experiments were performed in quadruplicate, and the mean values ± standard deviation are reported.

### Analysis of poly-P digestion using polyacrylamide gel electrophoresis (PAGE)

Reaction mixtures (100 μl) containing *E. coli* GPP, Rv1026, Rv0496 (MTB-PPX1) protein (5 μg) and 0.1 mM poly-P_130_ in 50 mM HEPES pH 6.8, 25 mM KCl, 1 mM MnCl_2_, were incubated at 37°C for 2 hours. Negative controls were analogously incubated: i) no added MnCl_2_, ii) no protein, iii) MBD protein (5 μg). Loading buffer (50 μl; 40% v/v glycerol +0.1% bromophenol blue) was added, and reaction mixtures were analyzed on 12% TBE-polyacrylamide gels and stained with toluidine blue as previously described [51]. Gels were visualized and bands quantified using a ChemiDoc XRS molecular imaging system with Quantity One v4.6.6 software (BioRad)

### Enzymatic preparation of ppGpp and pppGpp alarmones

The ppGpp and pppGpp nucleotides were synthesized enzymatically using recombinant EF-RelQ protein. Reaction mixtures (100 μl) contained EF-RelQ (5 μg) in 50 mM Tris-HCl pH 8.6, 100 mM NaCl, 10 mM MgCl_2_, 1 mM DTT, 1 mM ATP and 1 mM of GDP (to make ppGpp) or GTP (to make pppGpp), and were incubated for 1 h at 30°C. Synthesized pppGpp or ppGpp were purified by anion exchange chromatography (1 ml Resource Q column, GE Healthcare) on an AKTA purifier system (GE Healthcare). The eluent was monitored at 254 nm to detect and quantify nucleotide-containing fractions (see supplementary Figure 3). Identical runs using known concentrations (0–1 mM) of ATP, ADP, AMP, GTP, GDP, GMP, ppGpp and pppGpp were performed to enable unambiguous nucleotide identification and quantification. Fractions containing pure pppGpp or ppGpp were desalted by gel filtration chromatography on Sephadex G-10 (GE Healthcare), analogous to the method of Krohn and Wagner [42] and were characterized as described by Hardiman *et al.* [43].

### pppGpp hydrolysis assays

Rv0496 (MTB-PPX1), Rv1026, *E. coli* GPP or *E. coli* PPX protein (2 μg, ca. 0.1–0.2 μM) were incubated (30°C; 1–10 hours, as indicated in the text) with 0.1 mM pppGpp in 25 mM Tris-HCl pH 7.4, 0.5 mM DTT containing 1 mM MnCl_2_. Reaction products were analyzed and quantified by anion exchange chromatography as described above.

### ATP hydrolysis assays

ATP hydrolysis activities were determined by quantifying Pi release using the EnzChek phosphate assay kit, analogous to the method described above with minor modifications. Reactions (200 μl) contained Rv0496 (MTB-PPX1) or Rv1026 protein (1 μg), ATP (0.5–10 mM), 1 mM MnCl_2_, 4 mM (NH_4_)_2_SO_4_, in 50 mM Tris-HCl pH 7.4; were incubated at 37°C for 1 hour. Various concentrations of ATP (0.5, 1, 2, 5, 10 mM) were used to establish the steady-state kinetic constants for Rv0496 and Rv1026 proteins. Data were taken every minute, and *V_max_* and *K_m_* values were determined by fitting data to the Michaelis-Menten equation using Origin 6.0 (OriginLab). Experiments were performed in quadruplicate, reporting the mean values ± standard deviation.

### (p)ppGpp inhibition assays

#### Exopolyphosphatase activities

Real-time spectrophotometric phosphate release assays using the EnzChek assay reagents (Invitrogen) were performed at 37°C in 96-well plates, analogously to those described above; with Rv0496 (MTB-PPX1), EC-PPX or EC-GPP protein (1 μg, 0.1 μM), poly-P_130_ (125 μM) and the indicated amount of purified and desalted pppGpp or ppGpp (0–2 mM).

#### ATPase activities

EnzChek phosphate release assays analogous to those described above were performed at 37°C in 50 mM Tris-HCl pH 7.4, containing: Rv0496 or Rv1026 protein (1 μg, ca. 0.1 μM), 1 mM ATP, 1 mM MnCl_2_, 4 mM (NH_4_)_2_SO_4_; and the indicated amount of purified and desalted pppGpp or ppGpp (0–2 mM). For both sets of assays, *V_max_* values were determined and compared with corresponding values obtained in the absence of (p)ppGpp. The % decrease in *V_max_* values were reported as the mean ± standard deviation, obtained from three independent experiments, each performed in duplicate.

### Complementation assays for determining exopolyphosphatase and pppGpp hydrolase activities

#### Preparation of cell lysates

Stationary phase cultures of *E. coli* MG1655 (wild type) and CF6032 (*ΔgppA Δppkx*) [22] in 5 ml LB medium were expanded into 500 ml LB medium and incubated at 37°C until the early stationary phase (OD_600_ ca. 0.6–0.8). *Mycobacterium smegmatis* mc^2^155 was analogously cultured in Brain Heart Infusion (BHI) medium containing 0.05% Tween 20 (500 ml) at 37°C. Cells were harvested by centrifugation, washed and resuspended in 50 mM Tris-HCl (pH 7.4) +10% sucrose (20 ml). Lysozyme (250 μg/ml) was added to the resuspended *E. coli* MG1655 and CF6032 cell suspensions; which were frozen (liquid nitrogen), thawed, incubated at 37°C (4 mins), then chilled on ice (30 mins). Cells suspensions were (further) lysed and homogenized by sonication (1 s on, 9 s off; 20 cycles; ice cooling), before centrifugation (23,000× g; 15 mins; 2°C). Supernatants were decanted, and protein concentrations were determined by Bradford assay, before freezing aliquots in liquid nitrogen, for storage at −80°C.

#### pppGpp hydrolysis assays

Thawed cell lysates (2 μg protein) were incubated with 0.1 mM pppGpp in 25 mM Tris-HCl (pH 7.4), 0.5 mM DTT, 1 mM MnCl_2_ (150 μl) at 30°C for 2 hours. Analogous experiments were performed with the addition of 2 μg of MTB-PPX1, Rv1026, *E. coli* GPP, *E. coli* PPX or MBP (negative control) protein. Water (150 μl) was added, then reaction mixtures were immediately analyzed by anion exchange chromatography as described above.

#### Poly-P hydrolysis assays

Thawed cell lysates (2 μg protein) were incubated with 0.1 mM poly-P_130_ in 50 mM HEPES pH 6.8, 25 mM KCl, 1 mM MnCl_2_ (100 μl) at 37°C for 2 hours. Analogous experiments were performed with the addition of 2 μg of MTB-PPX1, Rv1026, *E. coli* GPP, *E. coli* PPX or MBP protein. Reaction mixtures were analyzed on TBE 12% polyacrylamide gels as described above.

## Supporting Information

Figure S1 **Alignment of selected PPX-GppA protein sequences.** The amino acid sequences of the Rv0496 (MTB-PPX1), Rv1026, *C. glutamicum* PPX1 (CG-PPX1, cg0488), *C. glutamicum* PPX2 (CG-PPX2, cg1115), *E. coli* GPP (EC-GPP), *E. coli* PPX (EC-PPX) and *A. aeolicus* Aq891 (Aq-PPX/GPPA) proteins were aligned using Clustal (Omiga 2.0, Oxford Molecular), manually trimmed and adjusted, then formatted using BOXSHADE 3.21 (K. Hofmann and M. Baron, http://www.ch.embnet.org/software/BOX_form.html). The positions of the conserved amino acids predicted to be bind Mn^2+^ or Mg^2+^ ions: Asp135 and Glu142 (MTB-PPX1) and Asp146 and Glu153 (Rv1026), are indicated with asterisks (*). The positions of conserved amino acids predicted to be directly involved in phosphoanhydride bond hydrolysis: Arg84 and Glu112 (MTB-PPX1) and Arg90 and Glu123 (Rv1026), are indicated with filled triangles (▴).
(TIF)

Figure S2 **Determination of protein purity and multimeric state.** Panels A–C: SDS polyacrylamide gels of purified recombinant proteins used in this study. ***Panel A***: *E. coli* GPP (EC-GPP; predicted molecular weight (MW): 51 kDa); ***Panel B***: *E. coli* PPX (EC-PPX; predicted MW: 53 kDa); and ***Panel C***: *E. faecalis* RelQ (EF-RelQ, predicted MW: 25 kDa). Benchmark protein ladder (Invitrogen) was included on each gel. ***Panel D***: Summary of light scattering and gel filtration results obtained for the MTB-PPX1, Rv1026, EC-GPP and EC-PPX proteins.
(TIF)

Figure S3 **Enzymatic synthesis of ppGpp and pppGpp using the EF-RelQ protein.** Reaction mixtures (100 μl) containing 5 μg EF-RelQ protein, 100 mM NaCl, 10 mM MgCl_2_, 1 mM DTT, 1 mM ATP and 1 mM GTP or GDP (for the synthesis of pppGpp or ppGpp, respectively) in Tris-HCl buffer (50 mM, pH 8.6), were incubated at 30°C for 0 or 60 minutes. Product mixtures were directly analyzed using anion exchange chromatography (1 ml ResourceQ column, AKTA-FPLC), eluting with a linear 25 mM to 1 M NaCl gradient in Tris-HCl (25 mM, pH 8.0). Chromatograms obtained are shown in Panels A–D. ***Panel A***: ATP + GTP, 0 minutes. ***Panel B***: ATP + GTP, 60 minutes. ***Panel C***: ATP + GDP, 0 minutes. ***Panel D***: ATP + GDP, 60 minutes incubation.
(TIF)

Figure S4 **Levels of pppGpp hydrolysis mediated by a cell free extract of** ***Escherichia coli*** **MG1655 (wild type strain) supplemented by MTB-PPX1, Rv1026,** ***E. coli*** **PPX or** ***E. coli*** **GPP proteins.** *E. coli* MG1655 cell lysate (2 μg total protein, see materials and methods) was incubated with 0.1 mM pppGpp in 25 mM Tris-HCl (pH 7.4), 0.5 mM DTT, 1 mM MnCl_2_ (150 μl) at 30°C for 2 hours. Products were analyzed by anion exchange chromatography (see materials and methods), with the chromatogram shown in Panel A. The elution profile of pppGpp and ppGpp under analogous conditions is shown in Panel B. Analogous experiments were performed with the addition of 2 μg of MTB-PPX1, Rv1026, *E. coli* GPP, *E. coli* PPX or maltose binding protein (MBP; negative control). The regions between 9 min and 14 mins (indicated with dashed lines in Panel A) on the five respective chromatograms obtained are shown in Panel C.
(TIF)

Figure S5 **Levels of pppGpp hydrolysis mediated by a cell free extract of** ***Escherichia coli*** **CF6032 (** ***ΔgppA Δppkx*** **mutant strain) supplemented by MTB-PPX1, Rv1026,** ***E. coli*** **PPX or** ***E. coli*** **GPP proteins.** *E. coli* CF6032 cell lysate (2 μg total protein, see materials and methods) was incubated with 0.1 mM pppGpp in 25 mM Tris-HCl (pH 7.4), 0.5 mM DTT, 1 mM MnCl_2_ (150 μl) at 30°C for 2 hours. Products were analyzed by anion exchange chromatography (see materials and methods), with the chromatogram shown in Panel A. The elution profile of pppGpp and ppGpp under analogous conditions is shown in Panel B. Analogous experiments were performed with the addition of 2 μg of MTB-PPX1, Rv1026, *E. coli* GPP, *E. coli* PPX or maltose binding protein (MBP; negative control). The regions between 9 min and 14 mins (indicated with dashed lines in Panel A) on the five respective chromatograms obtained are shown in Panel C.
(TIF)

Figure S6 **Levels of pppGpp hydrolysis mediated by a cell free extract of** ***Mycobacterium smegmatis*** **mc^2^155 supplemented by MTB-PPX1, Rv1026,** ***E. coli*** **PPX or** ***E. coli*** **GPP proteins.** *Mycobacterium smegmatis* mc^2^155 cell lysate (2 μg total protein, see materials and methods) was incubated with 0.1 mM pppGpp in 25 mM Tris-HCl (pH 7.4), 0.5 mM DTT, 1 mM MnCl_2_ (150 μl) at 30°C for 2 hours. Products were analyzed by anion exchange chromatography (see materials and methods), with the chromatogram shown in Panel A. The elution profile of pppGpp and ppGpp under analogous conditions is shown in Panel B. Analogous experiments were performed with the addition of 2 μg of MTB-PPX1, Rv1026, *E. coli* GPP, *E. coli* PPX or maltose binding protein (MBP; negative control). The regions between 9 min and 14 mins (indicated with dashed lines in Panel A) on the five respective chromatograms obtained are shown in Panel C.
(TIF)

Table S1 List of PCR primers used in this study.
(TIF)

Table S2 Determination of the mode by which pppGpp inhibits the ATPase activities of Rv1026. The *V_max_* values determined for each ATP concentration in the presence of 1 mM pppGpp, were compared with the *V_max_* values determined for analogous assays performed in the absence of pppGpp. The percentage decrease in *V_max_* values are reported in parentheses. *V_max_* values are reported as the mean value (μM/min) ± S.D.
(TIF)
