# Supplementary figures and images for: The Two PPX-GppA Homologues from Mycobacterium tuberculosis Have Distinct Biochemical Activities
Source: PLoS One. 2012 Aug 3;7(8):e42561. doi: 10.1371/journal.pone.0042561 (PMC3411833; doi:10.1371/journal.pone.0042561)

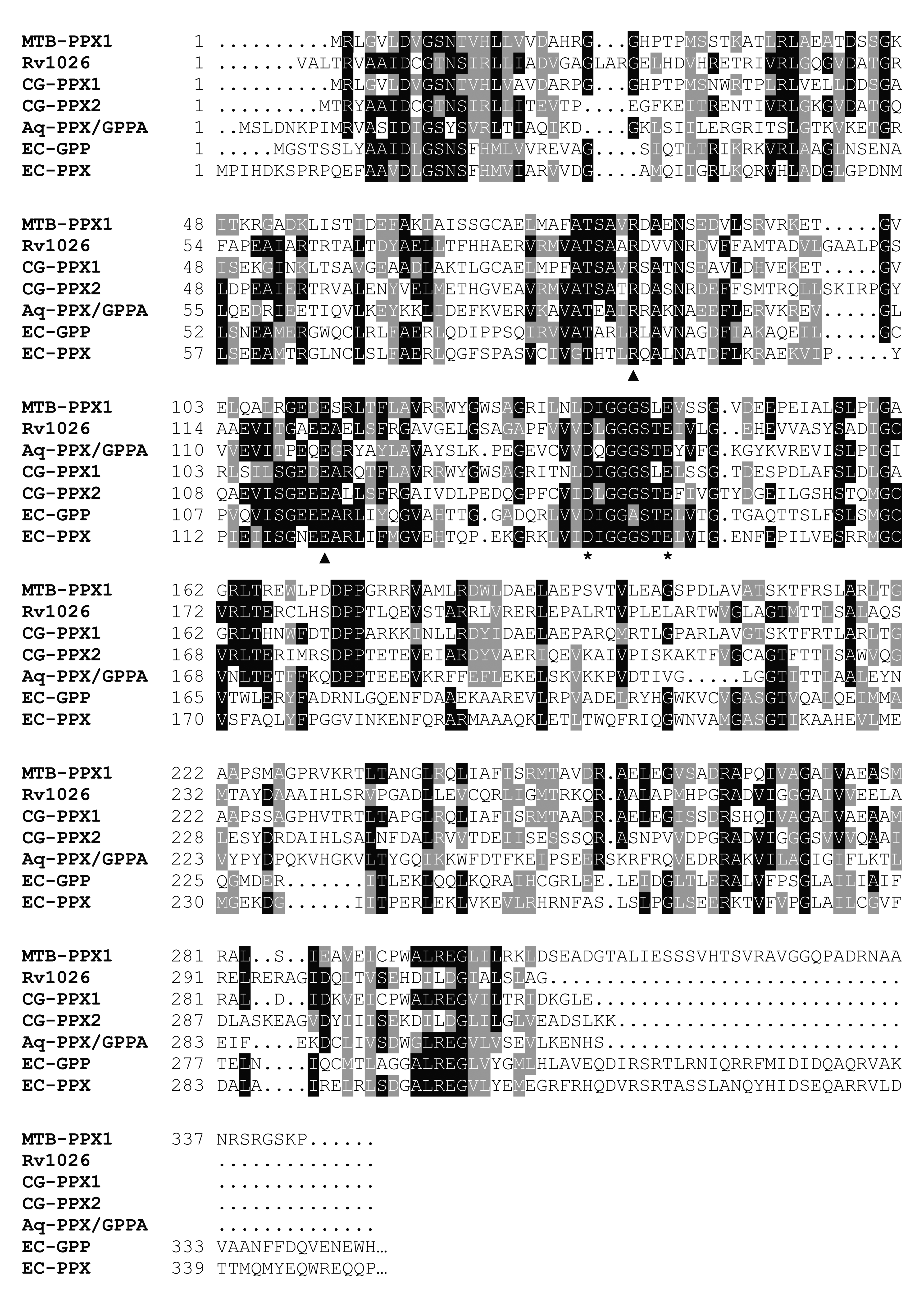

Supplement: Figure S1 — Alignment of selected PPX-GppA protein sequences. The amino acid sequences of the Rv0496 (MTB-PPX1), Rv1026, C. glutamicum PPX1 (CG-PPX1, cg0488), C. glutamicum PPX2 (CG-PPX2, cg1115), E. coli GPP (EC-GPP), E. coli PPX (EC-PPX) and A. aeolicus Aq891 (Aq-PPX/GPPA) proteins were aligned using Clustal (Omiga 2.0, Oxford Molecular), manually trimmed and adjusted, then formatted using BOXSHADE 3.21 (K. Hofmann and M. Baron, http://www.ch.embnet.org/software/BOX_form.html). The positions of the conserved amino acids predicted to be bind Mn2+ or Mg2+ ions: Asp135 and Glu142 (MTB-PPX1) and Asp146 and Glu153 (Rv1026), are indicated with asterisks (*). The positions of conserved amino acids predicted to be directly involved in phosphoanhydride bond hydrolysis: Arg84 and Glu112 (MTB-PPX1) and Arg90 and Glu123 (Rv1026), are indicated with filled triangles (▴). (TIF) [file pone.0042561.s001.tif]

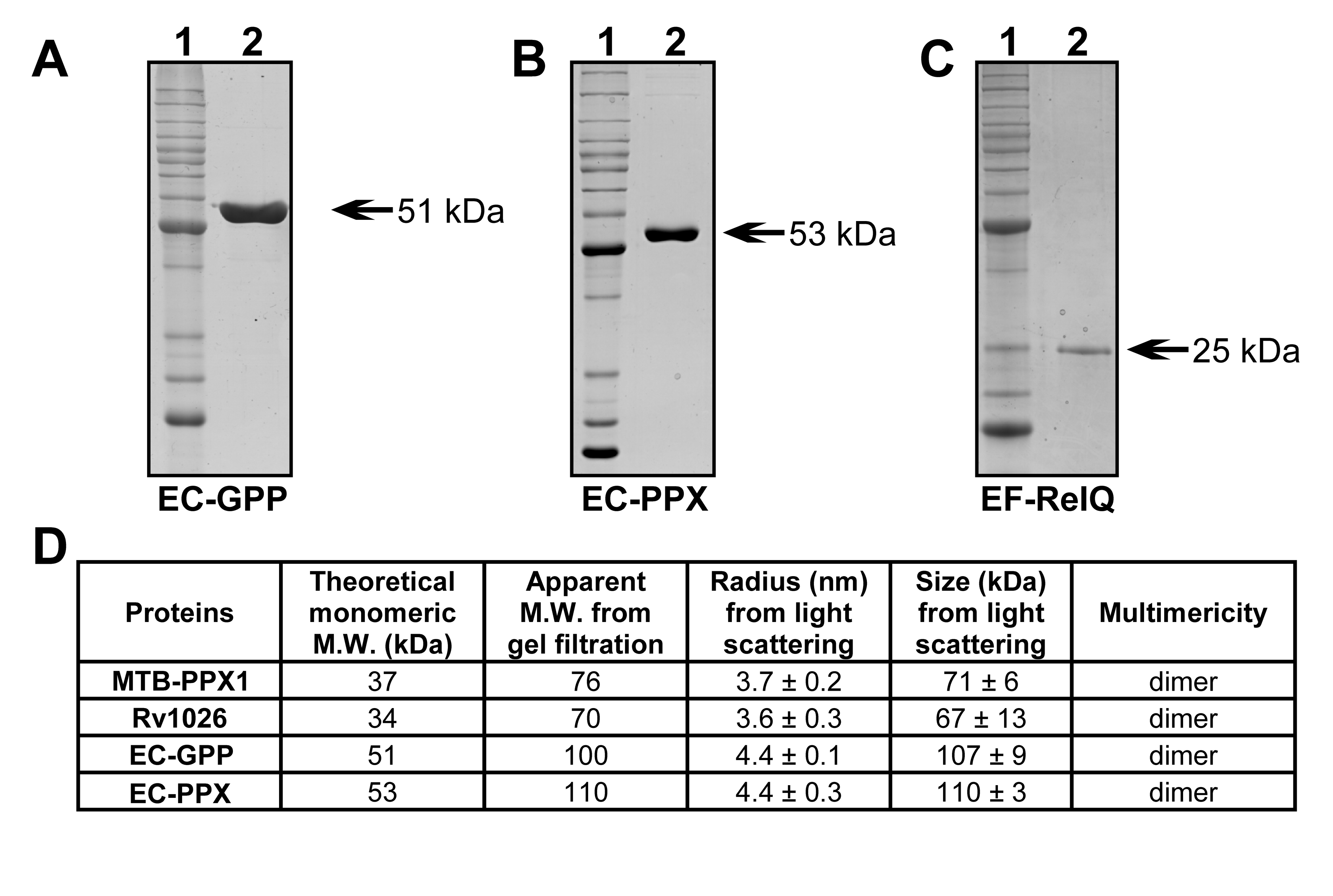

Supplement: Figure S2 — Determination of protein purity and multimeric state. Panels A–C: SDS polyacrylamide gels of purified recombinant proteins used in this study. Panel A: E. coli GPP (EC-GPP; predicted molecular weight (MW): 51 kDa); Panel B: E. coli PPX (EC-PPX; predicted MW: 53 kDa); and Panel C: E. faecalis RelQ (EF-RelQ, predicted MW: 25 kDa). Benchmark protein ladder (Invitrogen) was included on each gel. Panel D: Summary of light scattering and gel filtration results obtained for the MTB-PPX1, Rv1026, EC-GPP and EC-PPX proteins. (TIF) [file pone.0042561.s002.tif]

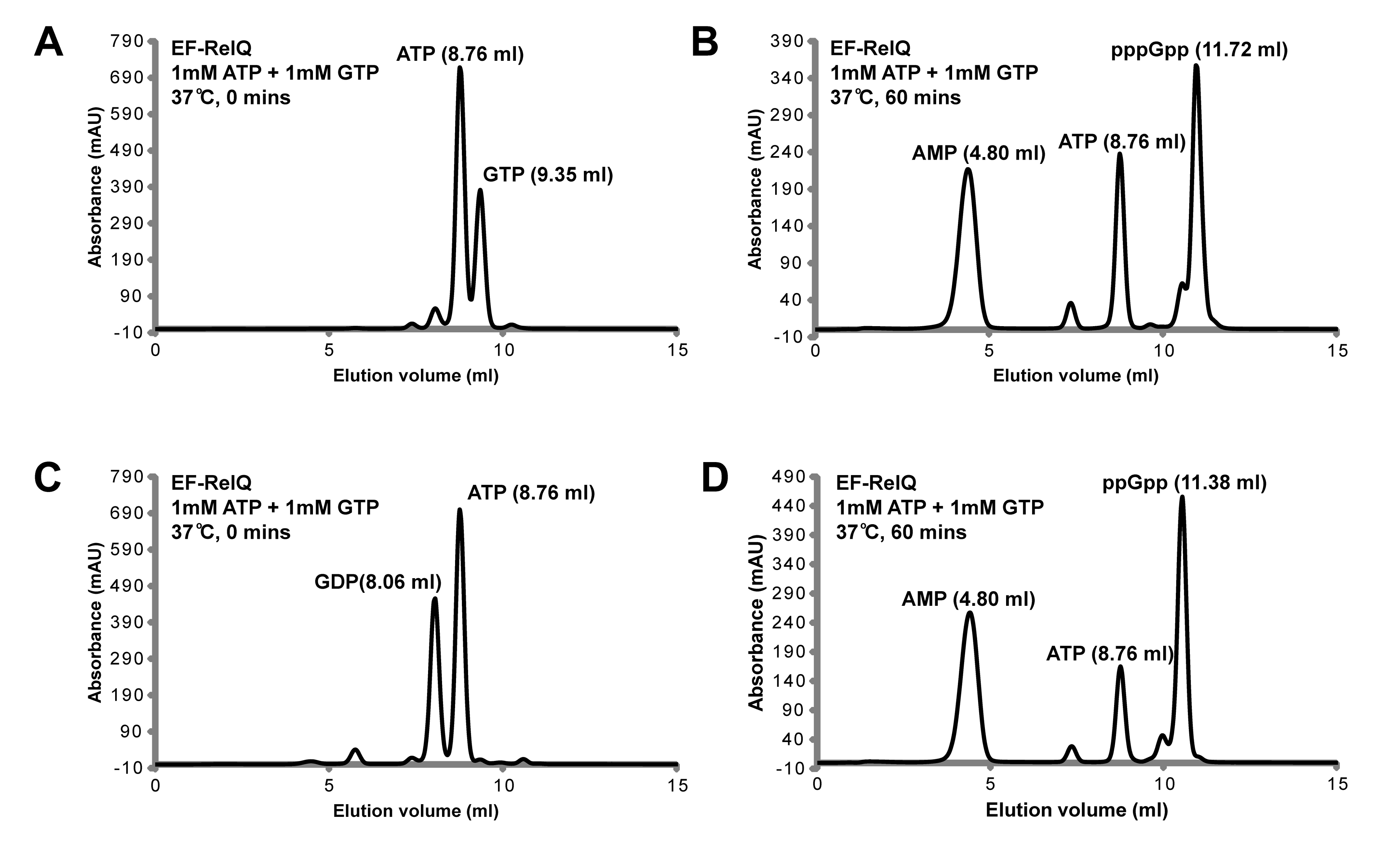

Supplement: Figure S3 — Enzymatic synthesis of ppGpp and pppGpp using the EF-RelQ protein. Reaction mixtures (100 μl) containing 5 μg EF-RelQ protein, 100 mM NaCl, 10 mM MgCl2, 1 mM DTT, 1 mM ATP and 1 mM GTP or GDP (for the synthesis of pppGpp or ppGpp, respectively) in Tris-HCl buffer (50 mM, pH 8.6), were incubated at 30°C for 0 or 60 minutes. Product mixtures were directly analyzed using anion exchange chromatography (1 ml ResourceQ column, AKTA-FPLC), eluting with a linear 25 mM to 1 M NaCl gradient in Tris-HCl (25 mM, pH 8.0). Chromatograms obtained are shown in Panels A–D. Panel A: ATP + GTP, 0 minutes. Panel B: ATP + GTP, 60 minutes. Panel C: ATP + GDP, 0 minutes. Panel D: ATP + GDP, 60 minutes incubation. (TIF) [file pone.0042561.s003.tif]

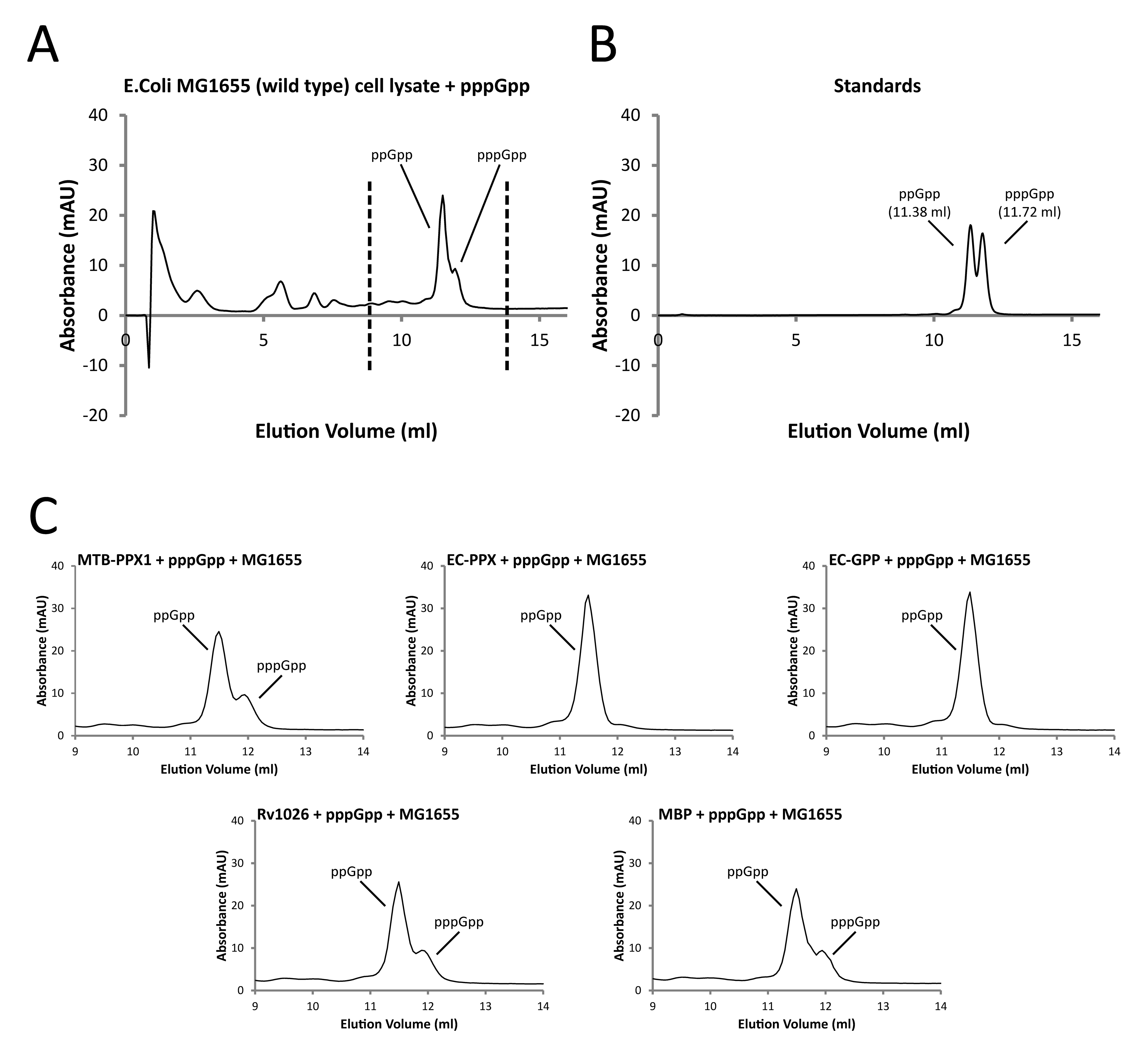

Supplement: Figure S4 — Levels of pppGpp hydrolysis mediated by a cell free extract of Escherichia coli MG1655 (wild type strain) supplemented by MTB-PPX1, Rv1026, E. coli PPX or E. coli GPP proteins. E. coli MG1655 cell lysate (2 μg total protein, see materials and methods) was incubated with 0.1 mM pppGpp in 25 mM Tris-HCl (pH 7.4), 0.5 mM DTT, 1 mM MnCl2 (150 μl) at 30°C for 2 hours. Products were analyzed by anion exchange chromatography (see materials and methods), with the chromatogram shown in Panel A. The elution profile of pppGpp and ppGpp under analogous conditions is shown in Panel B. Analogous experiments were performed with the addition of 2 μg of MTB-PPX1, Rv1026, E. coli GPP, E. coli PPX or maltose binding protein (MBP; negative control). The regions between 9 min and 14 mins (indicated with dashed lines in Panel A) on the five respective chromatograms obtained are shown in Panel C. (TIF) [file pone.0042561.s004.tif]

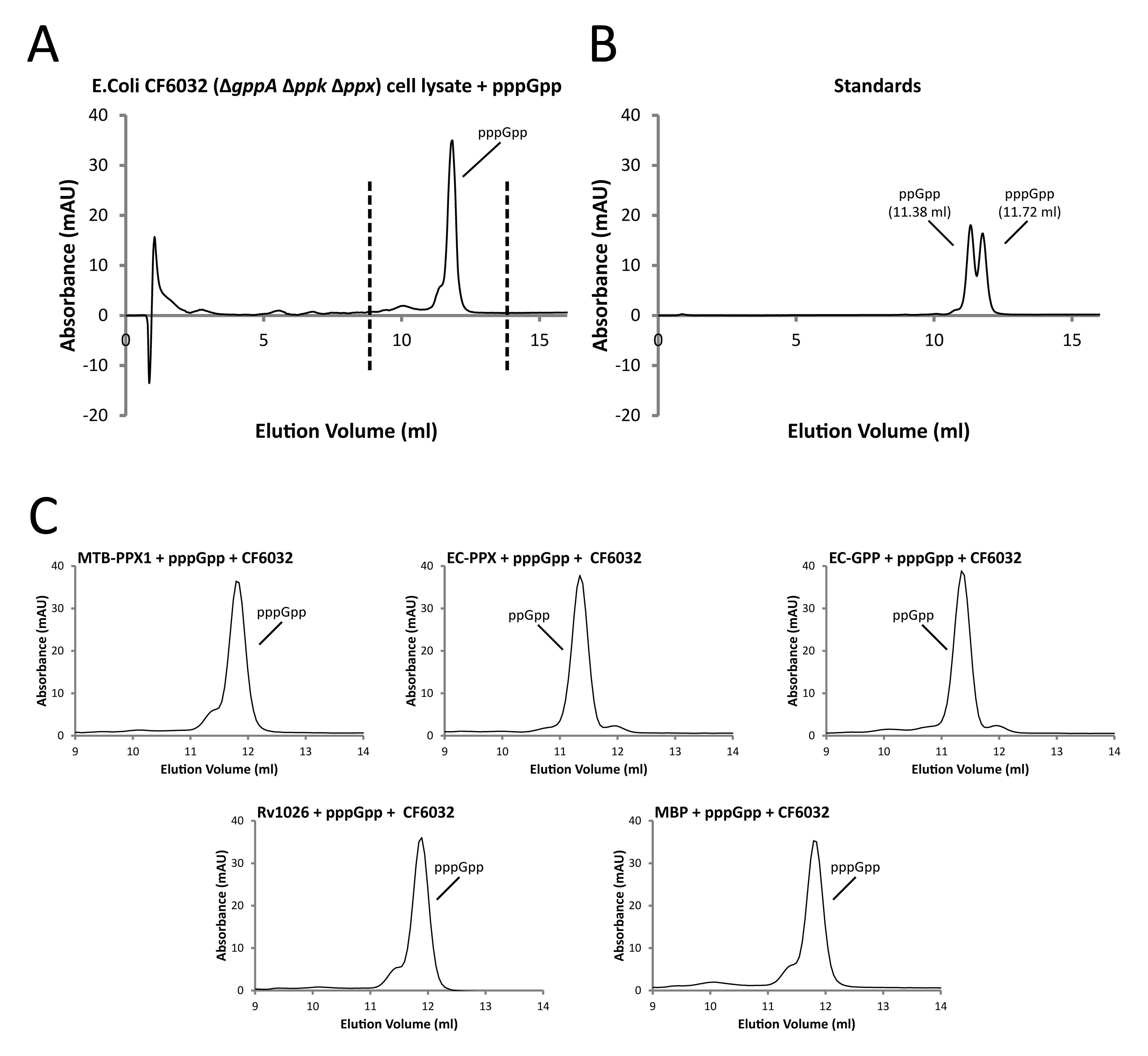

Supplement: Figure S5 — Levels of pppGpp hydrolysis mediated by a cell free extract of Escherichia coli CF6032 ( ΔgppA Δppkx mutant strain) supplemented by MTB-PPX1, Rv1026, E. coli PPX or E. coli GPP proteins. E. coli CF6032 cell lysate (2 μg total protein, see materials and methods) was incubated with 0.1 mM pppGpp in 25 mM Tris-HCl (pH 7.4), 0.5 mM DTT, 1 mM MnCl2 (150 μl) at 30°C for 2 hours. Products were analyzed by anion exchange chromatography (see materials and methods), with the chromatogram shown in Panel A. The elution profile of pppGpp and ppGpp under analogous conditions is shown in Panel B. Analogous experiments were performed with the addition of 2 μg of MTB-PPX1, Rv1026, E. coli GPP, E. coli PPX or maltose binding protein (MBP; negative control). The regions between 9 min and 14 mins (indicated with dashed lines in Panel A) on the five respective chromatograms obtained are shown in Panel C. (TIF) [file pone.0042561.s005.tif]

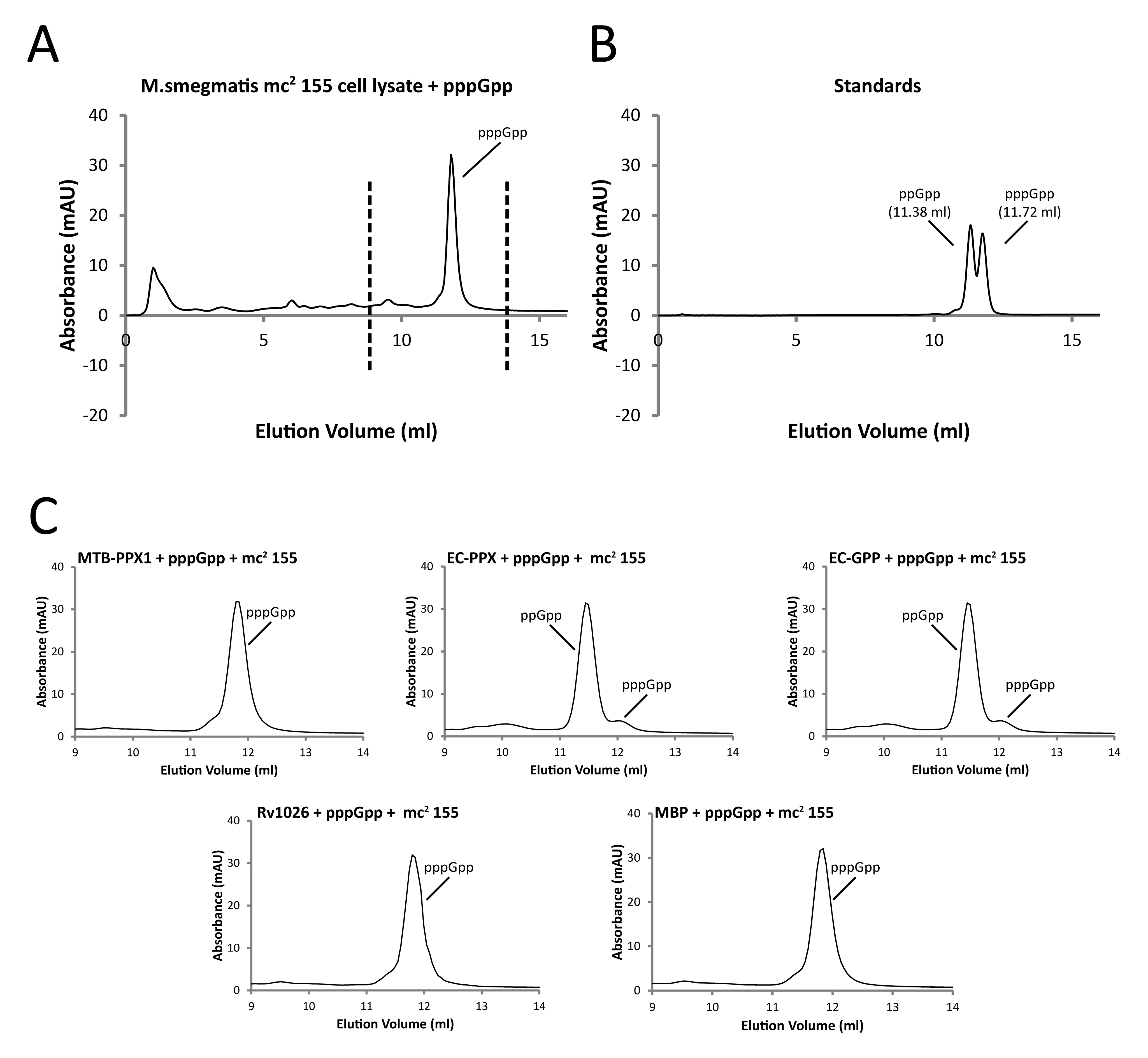

Supplement: Figure S6 — Levels of pppGpp hydrolysis mediated by a cell free extract of Mycobacterium smegmatis mc2155 supplemented by MTB-PPX1, Rv1026, E. coli PPX or E. coli GPP proteins. Mycobacterium smegmatis mc2155 cell lysate (2 μg total protein, see materials and methods) was incubated with 0.1 mM pppGpp in 25 mM Tris-HCl (pH 7.4), 0.5 mM DTT, 1 mM MnCl2 (150 μl) at 30°C for 2 hours. Products were analyzed by anion exchange chromatography (see materials and methods), with the chromatogram shown in Panel A. The elution profile of pppGpp and ppGpp under analogous conditions is shown in Panel B. Analogous experiments were performed with the addition of 2 μg of MTB-PPX1, Rv1026, E. coli GPP, E. coli PPX or maltose binding protein (MBP; negative control). The regions between 9 min and 14 mins (indicated with dashed lines in Panel A) on the five respective chromatograms obtained are shown in Panel C. (TIF) [file pone.0042561.s006.tif]

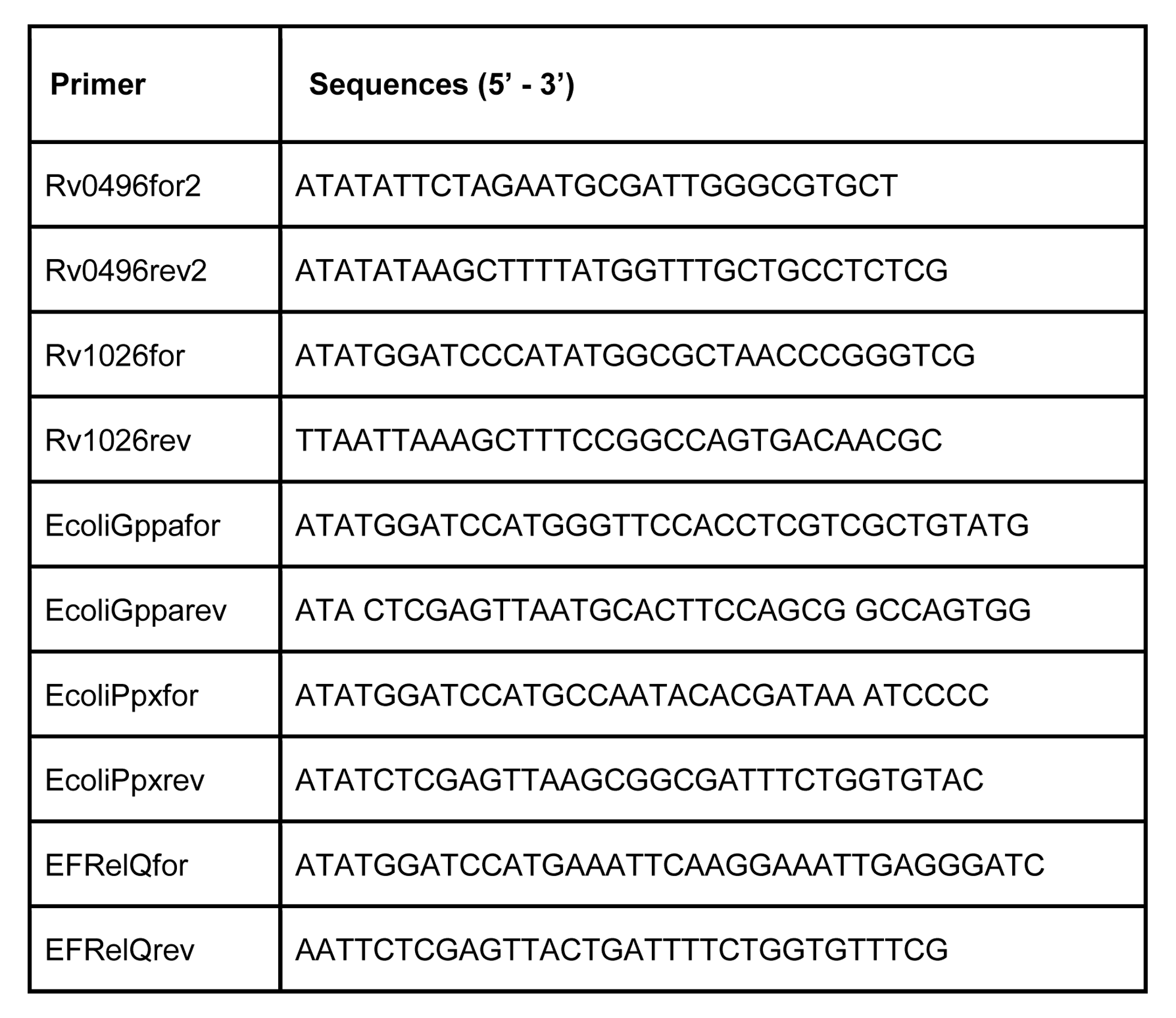

Supplement: Table S1 — List of PCR primers used in this study. (TIF) [file pone.0042561.s007.tif]

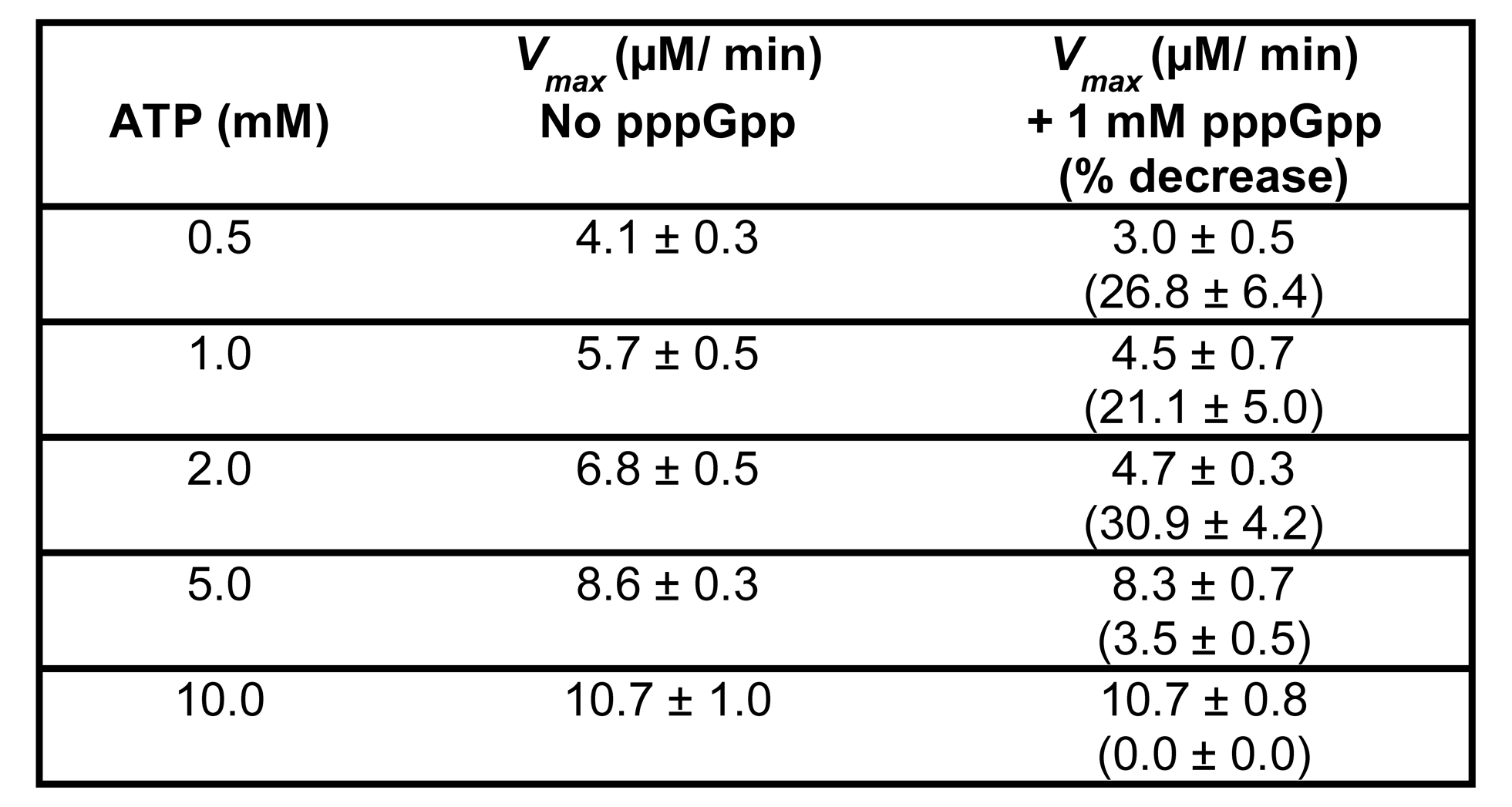

Supplement: Table S2 — Determination of the mode by which pppGpp inhibits the ATPase activities of Rv1026. The Vmax values determined for each ATP concentration in the presence of 1 mM pppGpp, were compared with the Vmax values determined for analogous assays performed in the absence of pppGpp. The percentage decrease in Vmax values are reported in parentheses. Vmax values are reported as the mean value (μM/min) ± S.D. (TIF) [file pone.0042561.s008.tif]
